# Gut Microbiome Alterations and Hepatic Metabolic Flexibility in the Gansu Zokor, *Eospalax cansus*: Adaptation to Hypoxic Niches

**DOI:** 10.3389/fcvm.2022.814076

**Published:** 2022-03-23

**Authors:** Jinyan Lin, Qi Yang, Juanjuan Guo, Meng Li, Zhiqiang Hao, Jianping He, Jingang Li

**Affiliations:** National Engineering Laboratory for Resource Development of Endangered Crude Drugs in Northwest China, Key Laboratory of the Ministry of Education for Medicinal Resources and Natural Pharmaceutical Chemistry, College of Life Science, Shaanxi Normal University, Xi’an, China

**Keywords:** Gansu zokor (*Eospalax cansus*), gut microbiome, hypoxia, *Ileibacterium*, hepatic metabolic flexibility

## Abstract

The Gansu zokor (*Eospalax cansus*), a typical subterranean rodent endemic to the Chinese Loess Plateau, spends almost its whole life in its self-constructed underground burrows and has strong adaptability to ambient hypoxia. Energy adaptation is the key to supporting hypoxia tolerance, and recent studies have shown that the intestinal microbiota has an evident effect on energy metabolism. However, how the gut microbiome of Gansu zokor will change in response to hypoxia and the metabolic role played by the microbiome have not been reported. Thus, we exposed Gansu zokors to severe hypoxia of 6.5% of O_2_ (6 or 44 h) or moderate hypoxia of 10.5% of O_2_ (44 h or 4 weeks), and then analyzed 16S rRNA sequencing, metagenomic sequencing, metagenomic binning, liver carbohydrate metabolites, and the related molecular levels. Our results showed that the hypoxia altered the microbiota composition of Gansu zokor, and the relative contribution of *Ileibacterium* to carbohydrate metabolism became increased under hypoxia, such as glycolysis and fructose metabolism. Furthermore, Gansu zokor liver enhanced carbohydrate metabolism under the short-term (6 or 44 h) hypoxia but it was suppressed under the long-term (4 weeks) hypoxia. Interestingly, under all hypoxia conditions, Gansu zokor liver exhibited enhanced fructose-driven metabolism through increased expression of the GLUT5 fructose transporter, ketohexokinase (KHK), aldolase B (ALDOB), and aldolase C (ALDOC), as well as increased KHK enzymatic activity and fructose utilization. Overall, our results suggest that the altered gut microbiota mediates the carbohydrate metabolic pattern under hypoxia, possibly contributing to the hepatic metabolic flexibility in Gansu zokor, which leads to better adaptation to hypoxic environments.

## Introduction

Rodents living in underground burrows face a formidable physiological challenge because these creatures must be forced to experience darkness, hypoxia, and hypercapnia (1–4). However, subterranean rodents are able to survive under this harsh environmental pressure in large part, because they have evolved various strategies, for instance, rewiring glycolysis and increasing fatty acid oxidation, to cope with the great challenge of high energy consumption in digging unpredictable food resources and maintaining the structure of the burrows (5–11). Hypoxia is a common environmental stressor for the subterranean rodents, so these animals also have evolved some solutions, such as a unique physiological function of sufficient antioxidant capacity that allows them to avoid or offset hypoxic damage (12–16). Notably, yet the current studies on the mechanism of hypoxia adaptation of the subterranean rodents have been mainly limited to a few species (i.e., naked mole rat, blind mole rat); furthermore, the existing studies rarely and simultaneously explore the intestinal microbiota composition. Therefore, investigating the effects of hypoxia on gut microbiota in Gansu zokor (*Eospalax cansus*), another typical subterranean rodent (17), and exploring many mechanisms of hypoxia tolerance are particularly important.

Gut microbiota, being emerged as a pivotal transducer of environmental factors, influences to exert effects on the heath and the metabolism of host animals (18–20), and its composition can be influenced by several environmental extremes, such as hypoxia, heat, and cold (21, 22). Hypoxia is a life-threatening environmental factor to most mammals, which leads to loss of consciousness or even death within minutes (23, 24). Indeed, even though hypoxia adaptation has been studied for several decades, the relationship between the gut microbiota and hypoxia has been receiving some attention only during the last few years. Studies have shown that hypoxia induces senescence of bone marrow mesenchymal stem cells *via* the decreased number of *Lactobacilli* (25); chronic intermittent hypobaric hypoxia might have anti-hypertension and anti-diabetes effects by altering the composition of the gut microbiota (26); exposure of low oxygen and high altitude hypoxia could change the intestinal microbial communities, which potentially may modulate metabolic processes in mice (27) and regulate the metabolic activity of the intestinal flora (28). However, almost all previous studies linking the gut microbiota with hypoxia were carried out in hypoxia-intolerant surface animals. Furthermore, studies on the gut microbiome in hypoxia-tolerant subterranean rodents are remarkably little; currently, a few studies showed that the gut of the blind mole-rat (*Spalax leucodon*) has a high abundance of longevity-linked *Muribaculaceae* bacteria (29), the naked mole-rat (*Heterocephalus glaber*) also possesses a unique gut microbiome composition, which supports its health and longevity (30), and this lends support to the beneficial relationship between the gut microbiota and the host (31); there are also some preliminary studies on the gut microbiome of plateau zokors (*Eospalax baileyi*) and Gansu zokors (*E. cansus*) (32, 33), but they were limited to the association between gut microbiota and different wild geographical locations or captivity conditions, without studying the effect of hypoxia on the intestinal flora of these special creatures.

It is established that hypoxic signaling and metabolism changes are highly interlinked, and the hypoxic microenvironment alters cellular metabolism (34). The liver is a highly active metabolic organ, which is responsible for metabolic homeostasis and is crucial for survival, but hypoxia could cause liver injury *via* oxidative stress mechanisms (35, 36); at the onset of liver damage, the glycometabolism disorders may occur (37). Surprisingly, some subterranean rodents can not only tolerate the hypoxia of their burrows for prolonged periods but can also live healthier and longer than the others (13, 38). Studies showed that *Spalax* downregulates the metabolic genes in the liver under hypoxia; this hypometabolism is a key to surviving the hypoxic or anoxic conditions (38). Furthermore, the naked mole-rat could mobilize its liver glucose in hypoxia to enhance carbohydrate metabolism, without causing metabolic acidosis (4). These findings indicate that metabolic remodeling of the liver tissue could be a potential favorable strategy against hypoxic damage. Nevertheless, studies regarding the underlying molecular mechanism of liver carbohydrate metabolism in the subterranean rodents *in vivo* are not enough, and even fewer studies have been reported on the liver metabolites under hypoxia.

**FIGURE 1:**
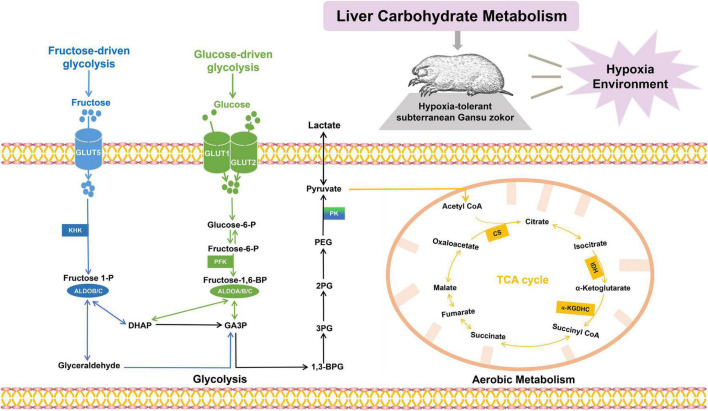
The carbohydrate metabolic pathways. Glucose enters hepatic cells *via* GLUT1 or GLUT2 and is converted by phosphofructokinase and aldolases (PFK; ALDOA, ALDOB, and ALDOC; shown in green); fructose enters hepatic cells *via* GLUT5 and is converted by ketohexokinase and aldolases (KHK; ALDOB, and ALDOC; shown in blue). Pyruvate enters the mitochondria and undergoes a series of oxidative reactions *via* citrate synthase (CS), isocitrate dehydrogenase, and α-ketoglutarate dehydrogenase (CS, IDH, and α-KGDHC; shown in orange).

In the present study, Gansu zokor was used to investigate the characteristics of hypoxia tolerance including gut microflora profiles and molecular mechanisms. Here, we hypothesized that hypoxia could alter the structure and composition of the gut microbiome in Gansu zokor, which mediates the rewired carbohydrate metabolism to support its hypoxia resistance. Thus, we comprehensively evaluated intestinal microflora, and the physiological and liver metabolism variables in Gansu zokors under normoxic (21% of O_2_) and hypoxic (10.5% of O_2_ or 6.5% of O_2_) conditions (Supplementary Figure 1). Fecal samples of Gansu zokors were collected for 16S rRNA gene sequencing and metagenomic sequencing, with the aim of revealing gut microbiome compositions and functions. Furthermore, we analyzed the liver metabolite profiling and detected the transcriptional and translational levels of carbohydrate metabolism-related genes in the liver, including the pathways of glycolysis (glucose-driven and fructose-driven glycolysis) and citrate cycle (Figure 1). In the glucose-driven glycolysis pathway, glucose enters hepatocytes *via* GLUT1 and GLUT2 transporters, and phosphofructokinase (PFK) which is a key enzyme that catalyzes the rate-limiting step from fructose-6-phosphate to fructose-1,6-bisphosphate (Fructose-1,6-BP) (39), and the Fructose-1,6-BP is metabolized by aldolases (40). In the fructose-driven glycolysis pathway, fructose enters the liver *via* GLUT5 transporter and is converted by ketohexokinase (KHK) to fructose-1-phosphate (F1P); then the F1P is directly metabolized into trioses *via* aldolase B (ALDOB) or aldolase C (ALDOC), thus bypassing the feedback inhibition of PFK regulatory block (9). The last step of glycolysis is catalyzed *via* pyruvate kinase (PK), which converts phosphoenopyruvate (PEP) into pyruvate; and the PKLR isoform is expressed specifically in the liver and the red blood cells (41, 42). The pyruvate generated from glycolysis is converted into acetyl-coenzyme A (CoA), citrate synthase (CS), a key metabolic enzyme in the tricarboxylic acid (TCA) cycle, and catalyzes the formation of citric acid and acetyl-CoA; besides, the isocitrate dehydrogenase (IDH) and α-ketoglutarate dehydrogenase (α-KGDHC) also play essential roles in the TCA cycle (43, 44). We then tested the activities of critical enzymes and the levels of glucose and fructose. The aim of these assays is to reveal the mechanisms of hypoxia tolerance in subterranean rodents.

## Materials and Methods

### Animals and Hypoxia Treatment

Gansu zokors are subterranean rodents that cannot be bred in captivity. In our study, these species (Supplementary Figure 2A, left) were captured in the field in Tongchuan, Shaanxi Province, China (35°12′N, 109°11′E). Zokors (Supplementary Figure 2A, right) were housed and fed in individual cages (47.5 × 35.0 × 20.0 cm) at room temperature and acclimated to the laboratory environment for 3 weeks prior to hypoxia exposure. All animals had *ad libitum* access to fresh carrots. This animal study was reviewed and approved by the Animal Management Committee and Ethical Review Committee of Experimental Animal Welfare, Shaanxi Normal University. Animals were treated and maintained in accordance with the China Wildlife Conservation Association.

We screened out some adult Gansu zokors referring to the literature of studies on age determination (45). Later, fifteen healthy adult female Gansu zokors (200–240 g) and ten healthy adult male Gansu zokors (220–260 g) were randomly divided into five groups (*n* = 5 replicates per group; 3 females and 2 males): (1) normoxia group (Norm: ambient O_2_ atmosphere); (2) severe hypoxia 6 h group (SH6h: 6.5% of O_2_ for 6 h); (3) severe hypoxia 44 h group (SH44h: 6.5% of O_2_ for 44 h); (4) moderate hypoxia 44 h group (MH44h: 10.5% O_2_ for 44 h) and (5) moderate hypoxia 4 weeks group (MH4w: 10.5% of O_2_ for 4 weeks). The hypoxia experiment was processed referring to a previous study (11). Fecal samples were collected at the end of experiments and snap-frozen in liquid nitrogen. Animals were anesthetized with pentobarbital sodium, and plasma samples from cardiac blood were collected after centrifugation (3,000 rpm, 10 min, 4°C). The middle part of the liver samples was removed, and one part of the liver was used for qRT-PCR, Western blotting, enzymatic activity, and for the determination of biochemical indicators; the other part of the liver was used for metabolite profiling; these detailed experimental procedures are available in the Supplementary Material. Fecal, plasma, and liver samples were stored at -80°C until further analyses.

### 16S rRNA Gene Amplicon Sequencing and Analysis

Fecal microbial DNA was extracted from all the samples using the E.Z.N.A.^®^ Soil DNA Kit (Omega Bio-tek, Norcross, GA, United States) according to the manufacturer’s protocols. Then, the hypervariable V3-V4 regions of the rRNA gene were amplified using universal primers (338F: 5′-ACT CCT ACG GGA GGC AGC AG-3′ and 806R: 5′-GGA CTA CHV GGG TWT CTA AT-3′) in the 20 μl of the mixture, which consisted 0.8 μl of each primer (5 μM), 4 μl of 5 × FastPfu Buffer, 2 μl of 2.5 mM dNTPs, 0.4 μl of FastPfu Polymerase, and 10 ng of DNA template under the following conditions: 95°C for 3 min, 27 cycles at 95°C for 30 s, 55°C for 30 s, and 72°C for 45 s, finishing with an extension step at 72°C for 10 min. The PCR products were extracted from 2% agarose gels and further purified and quantified using the AxyPrep DNA Gel Extraction Kit (Axygen Biosciences, Union City, CA, United States) and QuantiFluor™-ST (Promega, Madison, WI, United States), respectively. Purified amplicons were pooled in equimolar and sequenced on the Illumina MiSeq platform (Shanghai Majorbio Bio-Pharm Technology Co. Ltd., Shanghai, China) according to the standard protocols.

Raw 16S rRNA data were analyzed by the Quantitative Insights into Microbial Ecology (QIIME 1.17) software. Trimmed sequences were clustered into Operational Taxonomic Units (OTUs) at 97% sequence similarity cutoff using UPARSE,^1^ and chimeric sequences were identified and removed using UCHIME. The taxonomic assignment of OTUs was performed through the RDP Classifier^2^ against the SILVA database^3^ using a confidence threshold of 70%.

### Metagenomic Sequencing and Annotation

A total of eight fecal samples (Norm group: 2 females and 2 males; MH4w group: 2 females and 2 males) were selected for metagenomic sequencing. Total genomic DNA was extracted using the E.Z.N.A.^®^ Soil DNA Kit (Omega Bio-tek, Norcross, GA, United States) following the manufacturer’s instructions, the DNA concentration and purity were assessed using a Quantus Fluorometer (Promega, Madison, WI, United States) and NanoDrop 2000 spectrophotometer (Thermo Fisher Scientific Inc., Waltham, MA, United States), respectively. Agarose gel electrophoresis (1%) was used to monitor the DNA quality. Then, the microbial DNA was fragmented to an average size of approximately 300 bp using Covaris M220 (Covaris, Woburn, MA, United States) for paired-end library construction. The paired-end sequencing was performed on an Illumina HiSeq4000 sequencing platform (Illumina Inc., San Diego, CA, United States) at Majorbio Bio-Pharm Technology Co. Ltd. (Shanghai, China) according to the manufacturer’s protocols.

Raw sequence reads were trimmed to remove those containing ambiguous N bases or having a Phred score lower than 20 using Sickle.^4^ The clean raw reads were assembled by using the SOAPdenovo software^5^ to obtain contigs for the following prediction and annotation. Subsequently, open reading frames (ORFs) were conducted by MetaGeneAnnotator.^6^ On the cluster of orthologous groups of proteins (COGs), annotation of ORFs was performed using the Non-supervised Orthologous Groups (eggNOG) database using BLASTP (BLAST Version 2.2.28+) with an *e*-value cutoff of 1e^––5^, and the Kyoto Encyclopedia of Genes and Genomes (KEGG) pathway annotation was performed using BLASTP (BLAST Version 2.2.28+) against the KEGG database^7^ at an optimized *e*-value cutoff of 1e^–5^.

### Metagenomic Binning

For metagenomic binning, the clean metagenomic reads from the eight fecal samples were mapped to each assembly, and contigs with 1,000 bp minimum length were performed using CONCOCT^8^ and Metbat^9^ at default settings. Final bins were evaluated for completeness and contaminations by CheckM.^10^ Only those bins greater than or equal to 70% completeness and less than or equal to 10% contamination were retained for the following analysis. Annotation of the genomic bin was then performed using AMPHORA2.^11^

### Statistical Analysis

All sequencing analyses were performed using the Majorbio Cloud Platform.^12^ For 16S rRNA gene sequencing analysis, R Statistical Language based on OTUs at 97% sequence similarity was performed (46). Microbiome diversity was calculated using alpha diversity (ACE and Shannon index) and beta diversity [Hierarchical clustering tree and principal coordinate analyses (PCoA) based on Bray--Curtis distance] metrics. Statistical significance was determined with an analysis of similarities (ANOSIM). Kruskal--Wallis tests with multiple testing correction (FDR) were used to visualize the highly abundant taxa at the phylum and genus. Finally, the functional prediction was performed using PICRUSt2^13^ to predict the metabolic functions of KEGG pathways. Data were tested by one-way ANOVA followed by Tukey’s *post hoc* test or Student’s *t*-test, and the data were presented as mean ± standard deviation (SD). For metagenomic sequencing analysis, statistically significant differences in COG and KEGG categories were determined using linear discriminant analysis (LDA) effect size (LEfSe). Only LDA values greater than 2.5 and *P* < 0.05 were considered significantly enriched.

## Results

### Hypoxia Alters Fecal Microbiota Composition in Gansu Zokors

**FIGURE 2:**
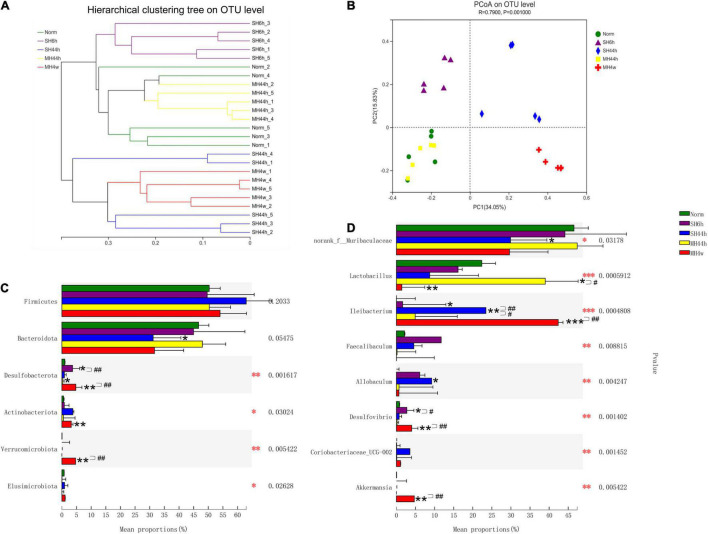
Beta-diversity of gut microbiota of Gansu zokors and relative abundances of microbial taxa. **(A)** Hierarchical cluster analysis of Bray–Curtis distances generated from taxa tables summarized at the OTU level. **(B)** Principal Coordinates Analysis represents the differences in the gut microflora among groups. Kruskal–Wallis test was used to examine the differences in the microbial taxa among the groups at the phylum level **(C)** and genus level **(D)**. The differences in the bacterial community composition among the different treatments by Kruskal–Wallis test (red asterisks). Black asterisks indicate the comparison between the control and hypoxia treatment groups by student’s *t*-test. Statistical symbols: *P < 0.05, **P < 0.01, and ***P < 0.001 between normoxia and hypoxia treatments; #*P* < 0.05 and ##*P* < 0.01 between two hypoxic groups under the same oxygen concentration or the same treatment duration.

To explore the effect of hypoxia on the intestinal microbiota of Gansu zokors, we first sequenced and analyzed the V3-V4 regions of the 16S rRNA gene. For twenty-five samples, the rarefaction curves of the Sobs index showed clear asymptotes, which confirmed sufficient depth of sequences (Supplementary Figure 2B). We assessed the microbial alpha diversity using the ACE and Shannon indices to estimate the richness and diversity, respectively. We found that the hypoxia treatments of SH44h and MH4w significantly reduced the richness, but there were no significant differences in the diversity after being exposed to hypoxia stress (Supplementary Figures 2C,D). Moreover, the results showed that the fecal microbiota of SH6h, SH44h, and MH4w groups was clearly separated from the normoxia group according to the hierarchical clustering tree (Figure 2A) and PCoA analyses (ANOSIM *R* = 0.7900, *P* = 0.001, Figure 2B). Notably, there was an overlap between normoxia and MH44h group in the microbiome structure, without clear separation (Figures 2A,B), which suggested that the hypoxia treatment of MH44h was relatively mild for Gansu zokor.

We next analyzed the fecal microbiota community composition in different treatments. The dominant phyla of the microbial community were Firmicutes and Bacteroidetes, which are accounted for more than 85% of the total sequences in all groups (Supplementary Figure 3). Compared to normoxia, SH6h and MH44h did not alter the two predominant phyla and Firmicutes/Bacteroidetes (F-B ratio); however, SH44h significantly decreased the relative abundance of Bacteroidota (*P* < 0.05), and both SH44h and MH4w increased the F-B ratio (*P* < 0.01 and *P* < 0.05, respectively Supplementary Figure 4B). We observed that the responses of gut microbiota to four hypoxia treatments showed different characteristics. On the top 6 phyla levels (Supplementary Figure 4A), when compared to normoxia, as shown previously, only SH44h significantly changed the predominant phylum; the abundance of Desulfobacterota was significantly increased in SH6h and MH4w, while it was significantly decreased in MH44h (*P* < 0.05); furthermore, significantly increased abundances of Actinobacteriota and Verrucomicrobiota were observed only in MH4w (*P* < 0.01) (Figure 2C). We then conducted the difference between the two hypoxia groups with equal oxygen concentration or equal hypoxia exposure time. In the comparison between SH6h and SH44h, we found that the abundance of Desulfobacterota was significantly decreased with the time of severe prolonged hypoxia; and in the comparison between MH44h and MH4w, we observed that the abundances of Desulfobacterota and Verrucomicrobiota were significantly increased with the time of moderate prolonged hypoxia (*P* < 0.01) (Figure 2C).

**FIGURE 3:**
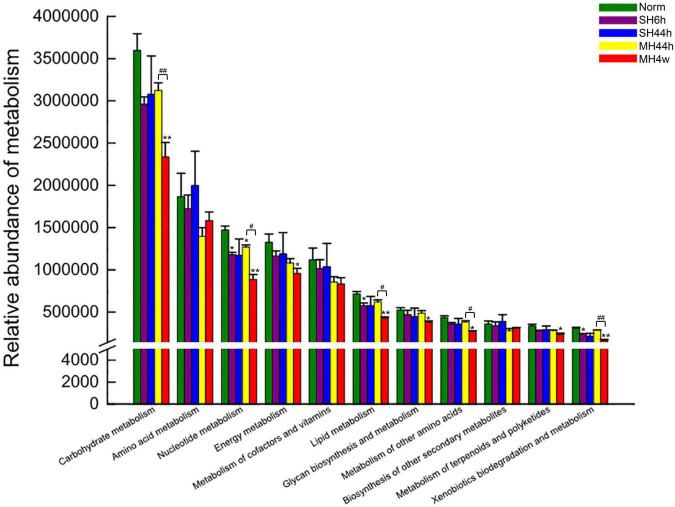
Functional prediction of metabolism between hypoxia treatment groups and normoxia. Bars indicate the mean ± SD. Statistical symbols: *P < 0.05 and **P < 0.01 between normoxia and hypoxia treatment; #*P* < 0.05 and ##*P* < 0.01 between two hypoxic groups under the same oxygen concentration or the same treatment duration.

Among the top 15 genera levels (Supplementary Figure 4C), we further detailed taxonomical shifts induced by different hypoxia treatments groups and found that when compared to normoxia, only SH44h significantly changed the relative abundances of *norank_f__Muribaculaceae* and *Allobaculum*; The MH44h showed little effect on the intestinal microbiota, which only significantly increased one genus of *Lactobacillus* (*P* < 0.05), while MH4w significantly decreased this genus level (*P* < 0.01) (Figure 2D). Interestingly, compared to the normoxia group, the genus, *Ileibacterium* was significantly increased in SH6h, SH44h, and MH4w groups, with a slight increase in the MH44h group, which seemed that *Ileibacterium* is sensitive to hypoxia stress in Gansu zokor; furthermore, the genus, *Desulfovibrio* was significantly increased in SH6h and MH4w groups (Figure 2D). We also observed that the phylum, Verrucomicrobiota contains only one genus of *Akkermansia* of fecal microbiota in Gansu zokor (Supplementary Table 1). Thus, like the Verrucomicrobiota phylum, the *Akkermansia* genus was virtually absent in SH6h, SH44h, and MH44h groups; however, it existed in the MH4w group with approximately 5% of the bacterial abundance, which was significantly overrepresented compared to its existence in normoxia (*P* < 0.01) (Supplementary Figure 2D and Supplementary Table 1). Similarly, we then analyzed the difference between the two groups of SH6h *vs.* SH44h, SH44h *vs.* MH44h, and MH44h *vs.* MH4w. In the comparison between SH6h and SH44h, we found that the genus, *Desulfovibrio* was significantly decreased with the time of severe prolonged hypoxia, and the abundance of *Ileibacterium* was significantly increased (Figure 2D). Compared to the SH44h group, the genus, *Ileibacterium* was significantly decreased in the MH44h group. In the comparison between MH44h and MH4w group, we observed that the *Lactobacillus* was significantly decreased with the time of moderate hypoxia, and the abundances of *Desulfovibrio* and *Akkermansia* were significantly increased (Figure 2D). We then predicted the metabolic functional profiling of microbial communities from its 16S rRNA sequencing data using the phylogenetic investigation of communities by the reconstruction of unobserved states (PICRUSt2^14^). Generally, the relative abundance of metabolism pathways in Gansu zokor showed a decreasing trend after being exposed to hypoxia, although not every hypoxia group reached statistical significance compared to normoxia (Figure 3). Furthermore, we found that the reduction in the MH4w group was more pronounced than those in the other three hypoxic groups; this treatment group significantly decreased the abundance of carbohydrate metabolism, nucleotide metabolism, energy metabolism, lipid metabolism, glycan biosynthesis and metabolism, metabolism of other amino acids, metabolism of terpenoids and polyketides, and xenobiotics biodegradation and metabolism (Figure 3, *P* < 0.05 or *P* < 0.01). Moreover, we then analyzed the difference between the two groups which have equal oxygen concentration or equal hypoxia exposure time. We found that in the comparison between MH44h and MH4w group, the abundances of carbohydrate metabolism, nucleotide metabolism, lipid metabolism, metabolism of other amino acids, and xenobiotics biodegradation and metabolism were significantly decreased with the time of moderate prolonged hypoxia (Figure 3, *P* < 0.05 or *P* < 0.01). Notably, of these metabolic pathways, the relative abundance of carbohydrate metabolism was at the highest level in all the groups; it is also worth noting that after being exposed to hypoxia, only the MH4w group dramatically reduced the carbohydrate metabolism, with a reduction up to 35.06% in relative abundance (*P* < 0.01).

### Metagenomic Analysis Revealed Different Carbohydrate Metabolism Functional Profiles Between Normoxia and Hypoxia, With Moderate Hypoxia 4 Weeks Group as a Hypoxic Representative

**FIGURE 4:**
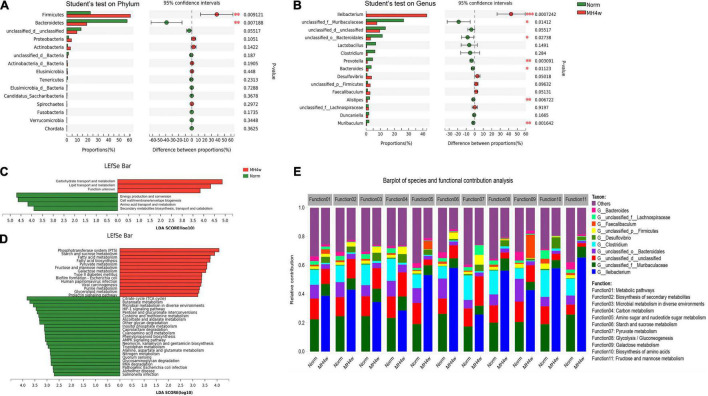
Statistical comparison of relative abundances of dominant microbial phyla **(A)** and genera **(B)** under normoxia and MH4w groups. Linear discriminant analysis (LDA) integrated with effect size (LEfSe) analysis of a cluster of orthologous groups of proteins (COG) **(C)** and the Kyoto Encyclopedia of Genes and Genomes (KEGG) **(D)** pathways between normoxia and MH4w groups (LDA score > 2.5, *P* < 0.05). **(E)** Relative contribution to carbohydrate metabolic pathways (top 11 in level 3) within the bacterial communities (top 10). Statistical symbols: *P < 0.05, ^**^P < 0.01, and ^***^P < 0.001.

Based on the above 16S rRNA sequencing findings, we focused our efforts on the effects of hypoxia on carbohydrate metabolism in Gansu zokor. We selected a gene set consisting of carbohydrate metabolism and then investigated the structure and functional profile of microbiota. A PCoA analysis showed the different fecal microbiota between the two groups in this gene set (Supplementary Figure 5A); besides, Firmicutes and Bacteroidetes are still the most dominant phyla (Supplementary Figure 5B). The MH4w treatment extremely increased the abundance of Firmicutes and significantly decreased the abundance of Bacteroidetes compared to normoxia (*P* < 0.01; Figure 4A); then on the genera levels, compared to normoxia, the genus, *Ileibacterium* was significantly increased after MH4w treatment (*P* < 0.001; Figure 4B), while other genera with significant differences, such as unclassified_f__*Muribaculaceae*, unclassified_o__*Bacteroidales* and *prevotella*, all showed a significant decrease in MH4w group (Figure 4B).

Furthermore, we analyzed the COG and KEGG functional profiles between the two groups. We first found that four functional COG categories were highly enriched in the normoxia group, including the energy production and conversion [C], cell wall/membrane/envelope biogenesis [M], amino acid transport and metabolism [E], and secondary metabolite biosynthesis, transport, and catabolism [Q]; in contrast, the MH4w group had more reads involved in the carbohydrate transport and metabolism [G] and lipid transport and metabolism [I] (Figure 4C). We then observed that 23 KEGG pathways (including citrate cycle, butanoate metabolism, microbial metabolism in diverse environments, and others) were significantly enriched in normoxia, and 14 KEGG pathways (including phosphotransferase system, starch and sucrose metabolism, fatty acid metabolism, and others) were significantly enriched in the MH4w group (Figure 4D). Notably, hypoxia caused more metabolism in the carbohydrate substrates, such as starch, sucrose, and fructose (Figure 4D).

To evaluate the functional contribution between the microbiome compositional changes and carbohydrate metabolic pathways, we next determined the taxonomic origin of carbohydrate metabolism-enriched functional attributes. As shown in Figure 4E, the top 10 differential bacterial taxa in terms of abundance were all associated with the same 11 most abundant pathways. We observed that the relative contribution of *Bacteroides*, unclassified_o__*Bacteroidales*, and unclassified_f__*Muribaculaceae* belonging to the phylum, Bacteroidetes in these pathways was decreased in the MH4w group compared to the normoxia. In contrast, the relative contribution of *Ileibacterium* and *Desulfovibrio* was increased after being exposed to hypoxia. Interestingly, the *Ileibacterium* in hypoxia showed even greater than 50% contribution in the pathways of amino sugar and nucleotide sugar metabolism (53.27%), starch and sucrose metabolism (58.17%), glycolysis/gluconeogenesis (56.28%), biosynthesis of amino acids (57.93%), and fructose and mannose metabolism (65.41%). Collectively, the genus, *Ileibacterium* played a crucial role in regulating the carbohydrate metabolism of Gansu zokor under hypoxia conditions.

### Binning Method Resolved Genomes From Metagenomic Datasets

In order to get a better idea about the genomes of fecal microorganisms of Gansu zokor, we used metagenomic binning to reconstruct genomes. The binning of metagenomic contigs resulted in the reconstruction of 430 genomes (bins less than 50% complete); among these, the taxonomic identification of 89 bins having completeness greater than or equal to 70% and contamination less than or equal to 10% were obtained (Supplementary Table 2). Of these high-quality bins, 15 bins were identified as Firmicutes, 48 bins as Bacteroidetes, 2 bins as Actinobacteria, 5 bins as Proteobacteria, and 1 bin as Spirochaetes. Together with the findings of the metagenomic analysis in carbohydrate metabolism, we then focused mainly on the top 10 bacterial taxa which contribute to this pathway. However, we found that only some metagenomic bins were identified at the genus level, and there were also some sequenced strains that did not match with the bins. Due to these reasons, we finally recovered 3 bacteria-related bins of the top 10 bacterial taxa, namely, *Bacteroidales*-related, *Clostridum*-related, and *Desulfovibrio*-related bins. Among these bins identified, 7 *Bacteroidales*-related bins, 6 *Clostridum*-related bins, and 2 *Desulfovibrio*-related bins were of high-quality (≥90% complete and ≤5% contamination; Supplementary Table 3). We then selected the best bins of each bacterium, Bin ID 118, Bin ID 33, and Bin ID 318, as the representative for further analysis.

**FIGURE 5:**
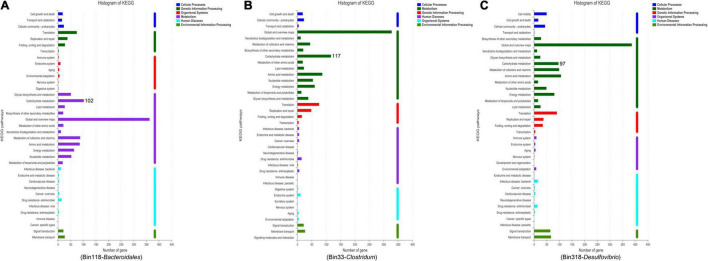
The KEGG functional pathway information based on *Bacteroidales*-related bin 118 **(A)**, *Clostridum*-related bin 33 **(B)**, and *Desulfovibrio*-related bin 318 **(C)**.

The *Bacteroidales*-related bin 118 had particularly high quality (98.49% completeness) and very low contamination (0.57%) with 52.05% GC; moreover, the *Clostridum*-related bin 33 (96.64% completeness) and *Desulfovibrio*-related bin 318 (93% completeness) were also very high-quality genomes, with 40.43 and 51.08% GC, respectively (Supplementary Tables 3, 4). Then functional categories were obtained after comparing the three genomes with KEGG reference databases. Interestingly, we found that a high number of genes were annotated in carbohydrate metabolism in bin 118 (*Bacteroidales*), bin 33 (*Clostridum*), and bin 318 (*Desulfovibrio*) (Figure 5), showing an important role in the carbohydrate metabolism pathway, which was consistent with the expected metagenomic analyses.

### Carbohydrate Metabolites of the Liver Revealed the Preferred Pathway to Meet an Energy Demand Under Hypoxia, Moderate Hypoxia 4 Weeks Group as a Hypoxic Representative

**FIGURE 6:**
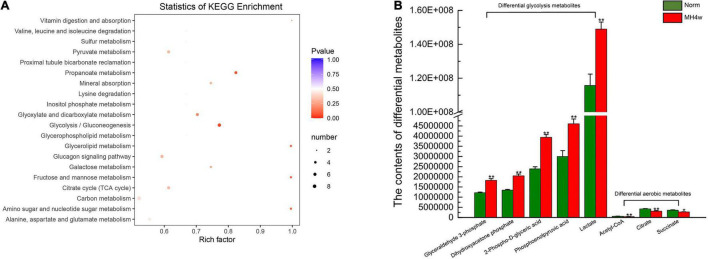
Liver differential metabolites between the two groups (Norm vs. MH4w). **(A)** Differential metabolite KEGG enrichment; the bigger the circle, the greater is the number of differential metabolites enriched in the pathway, the redder the circle, the more significant the enrichment. **(B)** Differential metabolites in glycolysis and aerobic metabolism. All data are presented as mean ± SD. Statistical symbols: *P < 0.05 and **P < 0.01.

We next investigated whether hypoxia affects the carbohydrate metabolism pathway in Gansu zokor. To identify the differences in metabolic profiles between normoxia and the MH4w group, principal component analysis (PCA) and partial least square discriminant analysis (PLS-DA) were performed, and we found that there were remarkable separations for Norm vs. MH4w (Supplementary Figures 6A,B). Furthermore, we assessed a total of 60 metabolites related to carbohydrate metabolism, and 29 metabolites were found significantly different (15 upregulated and 14 downregulated); the detailed information on these metabolites is presented in Supplementary Table 5. The statistics of KEGG enrichment for the differential metabolites in Norm vs. MH4w is shown in Figure 6A; according to the enrichment diagram, pathways, such as the propanoate metabolism, glycolysis/gluconeogenesis, glycerolipid metabolism, fructose and mannose metabolism, and amino sugar and nucleotide sugar metabolism were found to be significantly enriched for differentially expressed metabolites. Notably, 5 metabolites were significantly upregulated and 3 downregulated among those belonging to glycolysis and aerobic metabolism, respectively (Figure 6B). Taken together, these results suggest that hypoxia regulates carbohydrate metabolism, which may be better adaptability to a hypoxic environment.

### A Combined Analysis Revealed the Functional Correlation Between the Microbiome and Metabolites

**FIGURE 7:**
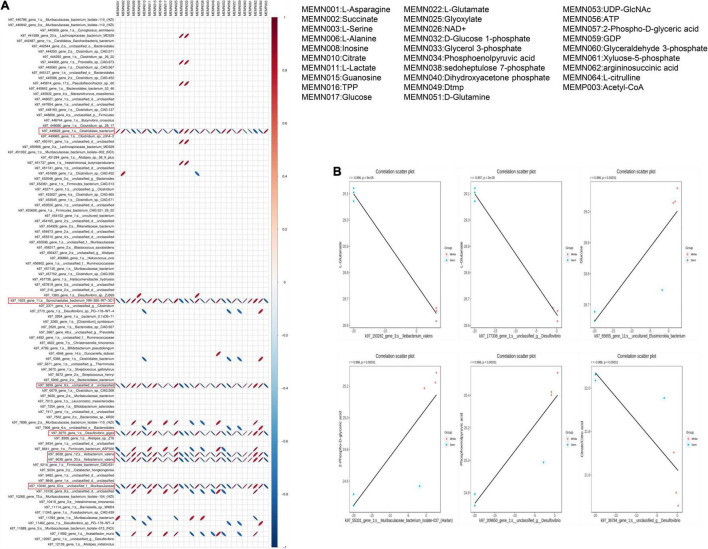
Correlation between differential metabolites and microorganisms. **(A)** The correlation heatmap of metabolites and microorganisms; red ellipses indicate positive relationship, blue ellipses indicate negative relationship (narrow ellipse = stronger correlation). **(B)** Scatter plots of some gut microflora–related metabolites, including L-Glutamate, glucose, 2-Phospho-D-glyceric acid, phosphoenolpyruvic acid, and citrate/citric acid.

To explore the potential correlations of the altered gut microbiome with metabolic profiling, the correlation was made using Pearson’s correlation coefficient. A heat map was drawn according to the top 20 differential metabolites with the highest VIP scores and differential microorganisms. We found that the *Clostridum_*bacterium, *Spirochaetales_*bacterium, *Desulfovibrio_*gigas, *Ileibacterium_*valens, and *Muribaculaceae* were all strongly correlated with the concerned metabolites (Figure 7A). The ellipses of positive correlations are shown in red right oblique and the negative correlations in blue left oblique (*P* < 0.05). Interestingly, the *Spirochaetales_*bacterium, *Desulfovibrio_*gigas, *Ileibacterium_*valens, and *Muribaculaceae* showed totally consistent correlations with metabolites, which was exactly the opposite of *Clostridum_*bacterium. These results suggest that there was a close correlation between these four gut bacteria and differential carbohydrate metabolic products; furthermore, the gut bacteria of *Spirochaetales_*bacterium, *Desulfovibrio_*gigas, *Ileibacterium_*valens, and *Muribaculaceae* might have a strong similarity in influencing the carbohydrate metabolites.

We then showed the functional correlation between the microbiome and metabolites by listing several metabolites that are highly correlated with bacteria (Figure 7B). For example, L-Glutamate negatively correlates with the *Ileibacterium* and *Desulfovibrio*, respectively. Glucose positively correlates with the Elusimicrobia and 2-Phospho-D-glyceric acid positively correlates with the *Muribaculaceae*. Likewise, phosphoenolpyruvic acid positively correlates with the *Desulfovibrio*, but citrate negatively correlates with the *Desulfovibrio*. In summary, hypoxia exposure induced changes in microbiome composition, which might substantially affect the metabolites, as proved by a certain correlation between them.

### Experimental Verification and Molecular Basis of Adaptive Responses to Hypoxia in Gansu Zokor

#### Quantitative Analysis of Carbohydrate Metabolism-Related Genes Expression in the Liver

**FIGURE 8:**
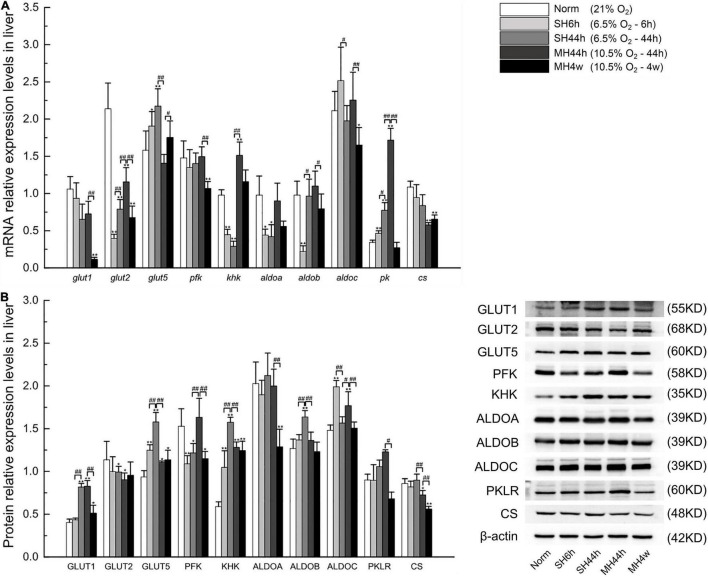
Transcription and translation level analyses of carbohydrate metabolism-related transporters and enzymes in Gansu zokor. **(A)** Gene expressions graph, **(B)** protein expression graph, and the representative images of at least three separate blots on the right side of it showing the expression of the proteins. All data are presented as mean ± SD. Statistical symbols: *P < 0.05 and **P < 0.01 between normoxia and hypoxia treatment; #*P* < 0.05 and ##*P* < 0.01 between two hypoxic groups under the same oxygen concentration or the same treatment duration.

To better understand the pattern of carbohydrate metabolism following hypoxic exposure, we first analyzed the expression of related genes in Gansu zokor under the conditions of hypoxia compared to those under normoxia by qRT-PCR using the primers shown in Supplementary Table 6. In the glucose-driven glycolysis pathway, we observed that the *glut1* mRNA expression was significantly decreased in the MH4w group (*P* < 0.01) (Figure 8A), whereas the GLUT1 protein expression was significantly increased in SH44h and two moderate hypoxia groups (Figure 8B). All hypoxia conditions induced decreases in the *glut2* mRNA expression, but this differential expression in GLUT2 protein expression was found only in SH44h and MH44h groups (Figures 8A,B). The *pfk* mRNA expression was decreased in SH6h, SH44h, and MH4w groups, with the protein levels corresponding to the mRNA levels (Figures 8A,B). In the fructose-driven glycolysis pathway, the *glut5* mRNA expression was significantly increased in the three groups of SH6h, SH44h, and MH4w, and at the protein expression level, this differential expression was found in all hypoxia conditions (Figures 8A,B). Interestingly, although the expression level of *khk* mRNA was significantly increased only in the MH44h group (*P* < 0.01) (Figure 8A), its protein expression level was significantly increased in all the hypoxia groups (*P* < 0.01) (Figure 8A). Moreover, there was no significant increase of aldolases (*aldoa*, *aldob*, and *aldoc*) in the mRNA expression of all the hypoxia groups, but the protein expression levels of ALDOB and ALDOC were significantly increased in some hypoxia groups (*P* < 0.01) (Figures 8A,B). The *pk* gene expression was strongly upregulated after hypoxic treatments of SH6h, SH44h, and MH44h (*P* < 0.01) (Figure 8A), but it did not translate into increased protein levels (Figure 8B). The *cs* mRNA expression was significantly decreased in the two moderate hypoxia groups, and its protein levels were consistent with the mRNA levels, showing a high degree of consistency (Figures 8A,B).

The hypoxia treatments in our study were set at equal oxygen concentration or equal hypoxia exposure time. Then, we conducted the difference between the two groups of SH6h *vs.* SH44h, SH44h *vs.* MH44h, and MH44h *vs.* MH4w. In the comparison between the SH6h and SH44h groups, we found that the mRNA levels of *glut2*, *aldob*, and *pk*, and the protein levels of GLUT1, GLUT5, KHK, and ALDOB were significantly increased with the time of severe prolonged hypoxia (Figures 8A,B), and the *aldoc* expression in both the mRNA and its protein levels were significantly decreased (Figure 8B). Compared to the SH44h group, the mRNA levels of *glut2*, *khk*, and *pk*, and the protein levels of PFK and ALDOC were significantly increased in the MH44h group; but the mRNA level of *glut5* and the protein levels of GLUT5, KHK, ALDOB, and CS were significantly decreased in the MH44h group (Figures 8A,B). In the comparison between MH44h and MH4w groups, we observed that the mRNA levels of *glut1*, *glut2*, *pfk*, *aldob*, *aldoc*, and *pk*, and the protein levels of GLUT1, PFK, ALDOA, ALDOC, PKLR, and CS were significantly decreased with the time of moderate prolonged hypoxia (Figures 8A,B), and only the expression level of *glut5* mRNA was significantly increased (Figure 8A). Overall, under the oxygen concentration of 6.5% O_2_, the carbohydrate metabolism-related genes in Gansu zokor were upregulated as treatment time increased; interestingly, under the oxygen concentration of 10.5% O_2_, these genes were downregulated as treatment time increased; besides, at the same treatment duration of 44 h, Gansu zokor showed larger variations in carbohydrate metabolism-related genes expression under different oxygen concentration treatments (6.5 or 10.5% O_2_).

#### Quantitative Analysis of Carbohydrate Metabolism-Related Enzymatic Activity

**FIGURE 9:**
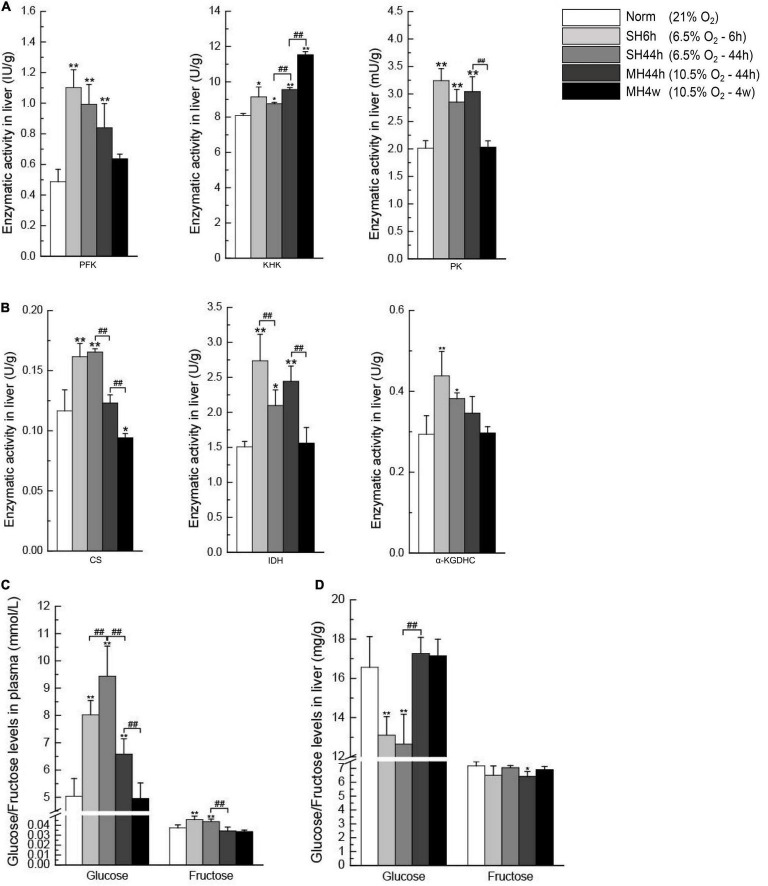
Enzymatic activity analyses of carbohydrate metabolism and glucose and fructose levels in the plasma and the liver. The activities of PFK, KHK, and PK **(A)** and CS, IDH, and α-KGDHC **(B)**. Plasma glucose and plasma fructose levels **(C)**. Liver glucose and liver fructose levels **(D)**. All data are presented as mean ± SD. Statistical symbols: *P < 0.05 and **P < 0.01 between the normoxia and hypoxia treatment; ##*P* < 0.01 between two hypoxic groups under the same oxygen concentration or the same treatment duration.

In the glycolytic pathway, PFK, KHK, and PK are key kinases since they perform irreversible steps (Figure 1); we observed that SH6h, SH44h, and MH44h significantly increased the enzymatic activity of the three important regulatory enzymes in Gansu zokor liver, the MH4w only significantly increased the KHK enzymatic activity (Figure 9A). In the process of TCA cycle, CS, IDH, and α-KGDHC are key enzymes; they mediate the irreversible rate-limiting steps (Figure 1); we found that SH6h and SH44h significantly increased the activity of these three enzymes; MH44h significantly increased the IDH enzymatic activity and MH4w significantly decreased the activity of CS (Figure 9B).

Similarly, we then analyzed the difference between the two groups which have equal oxygen concentration or equal hypoxia exposure time. Compared to the SH6h group, only the IDH enzymatic activity was significantly decreased in the SH44h group (*P* < 0.01) (Figure 9B). Compared to the SH44h group, the activity of KHK was significantly increased, and CS was significantly decreased in the MH44h group (*P* < 0.01) (Figures 9A,B). Compared to the MH44h group, the activity of PK, CS, and IDH was significantly decreased in the MH4w group (*P* < 0.01) (Figures 9A,B); however, the KHK enzymatic activity was significantly increased in the MH4w group (*P* < 0.01) (Figure 9A). Thus, we found differences in the enzymatic activity of carbohydrate-related metabolism of Gansu zokor liver under different hypoxia conditions.

#### Changes in Glucose and Fructose Levels in Hypoxia

Glucose and fructose are fuel sources for carbohydrate metabolism. To evaluate whether hypoxia affects these sugar contents, we detected their levels in the plasma and the liver. We first observed that the two severe hypoxia groups significantly increased the plasma glucose and plasma fructose levels compared to normoxia; besides, the plasma glucose level was also significantly increased in the MH44h group (*P* < 0.01) (Figure 9C). In contrast, compared to normoxia, the levels of hepatic glucose and hepatic fructose were significantly decreased in some hypoxia groups, and all hypoxia conditions did not significantly increase the levels of glucose or fructose in the liver (Figure 9D). We then conducted the difference between the two hypoxia groups of having the equal concentration or equal time; notably, we found that the level of plasma glucose in SH44h was upregulated compared to SH6h or MH44h; conversely, the hepatic glucose content in SH44h was downregulated compared to these two groups (Figures 9C,D).

## Discussion

In the present study, for the first time, we investigated the effect of hypoxia on the gut microbiota in Gansu zokor. Moreover, we explored the metabolism pattern of glucose-driven glycolysis, fructose-driven glycolysis, and the TCA cycle in hypoxia. Together, these studies reveal the further mechanisms underlying the hypoxia tolerance in the subterranean rodents. Overall, our study yielded several important findings: (1) hypoxia exposure induced a change in the gut microbiota composition in Gansu zokor, especially in the SH44h and MH4w groups. (2) The genus, *Ileibacterium* played a crucial role in regulating the carbohydrate metabolism of Gansu zokor under hypoxia conditions, particularly in the fructose and mannose metabolism pathway. Likewise, *Bacteroidales, Clostridum*, and *Desulfovibrio* also appeared to play a role in affecting carbohydrate metabolism based on the analysis of both metagenomic and related bin genomes. (3) The molecular signatures of fructose metabolism, GLUT5, and KHK were upregulated at protein levels by hypoxia stress, as well as the KHK enzymatic activity, which showed that all the hypoxia conditions increased the fructose-driven glycolysis in the Gansu zokor liver. (4) Overall, the MH4w group decreased all the carbohydrate metabolism and the related genes levels, and the three short-term (6 or 44 h) hypoxia increased these genes levels, including the enzymatic activities in carbohydrate metabolism. Based on these findings, we postulated that Gansu zokors harbor a unique gut microflora in response to hypoxia of the subterrestrial environment; moreover, Gansu zokors may have different carbohydrate metabolism patterns under different hypoxia conditions, showing a high degree of metabolic flexibility reacting to hypoxia stresses.

### Hypoxia Exposure (Severe Hypoxia 44 h Group and Moderate Hypoxia 4 Weeks Group) Significantly Alters the Gut Microbiota Diversity and Composition in Gansu Zokor

Organism-associated microbial communities have effects on the physiology of their hosts, critically contributing to host adaptation to the habitat environment (47). Some wild rodents inhabit underground burrows with dynamic hypoxia conditions, and these creatures can survive normally in a stressful environment (4, 9). Unfortunately, until now, no studies have found the composition of the gut microbiota in hypoxia-tolerant subterranean rodents under hypoxia. To fill this gap, we exposed Gansu zokors to hypoxia conditions that might occur in their wild habitats (48). As we previously postulated, hypoxia could alter the structure and composition of the gut microbiome in Gansu zokor; this change may help Gansu zokors adapt to the hypoxia environment of underground burrows.

Whether in normoxia or hypoxia, the gut bacteria of the two predominant phyla of Gansu zokors were Firmicutes and Bacteroidetes; this is consistent with the studies on naked mole-rat, blind mole-rat, and plateau zokors under normoxia (29, 33, 49), indicating that Gansu zokor has the same dominant phylum with other wild subterranean rodents. Moreover, an increased Firmicutes/Bacteroidetes (F-B) ratio under hypoxia conditions of SH44h and MH4w indicates that these two treatments have a higher impact on the gut microbiota; meanwhile, the SH44h and MH4w groups also showed higher F-B ratio than other hypoxia groups of having the equal concentration or time, which is also embodied in the ecological diversity metrics analyses (alpha diversity and beta diversity). The F-B ratio is an important attribute of the microbial communities (50); several studies recently reported that the increased F-B ratios were related to obesity, obstructive sleep apnoea syndrome, and metabolic syndrome (51–53). However, the consequences of the increased F-B ratio are not clearly established. For example, high-fat intake increases the F-B ratio and contributes to inflammation, while exercise similarly increases this ratio and decreases inflammation (54, 55). Thus, we cannot determine the effect of increased F-B ratio on Gansu zokor after being exposed to SH44h and MH4w. Here, we can only speculate that the changes in the gut microbiota may be a response to the hypoxic environment, which helps Gansu zokor adapt to hypoxia. Nevertheless, how the increased F-B ratio affects hypoxia response is not well understood, which needs to be further studied to clarify.

As described previously, a higher abundance of the *Ileibacterium* genus was observed in Gansu zokor after hypoxia exposure. However, no such finding has been reported in other studies on the effects of hypoxia on the gut microbiota; as far as we are aware, this is the first time that this phenomenon is observed through exploring the alterations of the intestinal flora in the wild subterranean rodent under hypoxia. The *Ileibacterium* genus was first reported in 2017 (56); it belongs to the family, *Erysipelotrichaceae*. Some studies indicated that the metabolism disorder induced by chronic alcohol consumption caused a decrease in the relative abundance of *Ileibacterium*, and the restoration of *Ileibacterium* might mitigate the hyperlipidemia (57, 58); while others indicated that the *Ileibacterium* genus was positively correlated with serum lipid levels and metabolic disorders (59, 60); moreover, a study postulated that this bacterium might be related to energy expenditure (61). Apparently, inconsistent analysis results have emerged from different studies, which may be associated with the different experimental designs and different analyses. Regrettably, there is still no literature that has reported the relationship between the *Ileibacterium* genus and hypoxia. Here, the postulation from the study by den et al. seems to be more accepted in our study; namely, we speculate that the increased *Ileibacterium* genus after hypoxia is most likely related to the energy metabolism regulation; this certainly requires further investigation on microflora functionality. Furthermore, it is also worth noting that although the *Akkermansia* genus is virtually absent in Gansu zokor under normoxia and a brief period of hypoxia (6 or 44 h), this genus shows a dramatic increase with the hypoxia treatment for 4 weeks, which suggests that the *Akkermansia* genus may exert important function in prolonged hypoxia acclimation. Notably, *Akkermansia* is a beneficial bacterium, which has been found to play a vital role in ameliorating metabolic disorders and has potential anti-inflammatory properties (62–66). Thus, we suggest that the increasing *Akkermansia* would be helpful to ensure healthy Gansu zokors in their subterranean burrows, which is a manifestation of hypoxia adaptation.

Additionally, maintaining energy balance is one of the keys to hypoxia tolerance (67, 68); and the gut microbiota could influence host energy metabolism through a series of mechanisms (69, 70). In this study, functional prediction in intestinal microbiota revealed that hypoxia reduces the relative abundance of metabolic pathways, especially in carbohydrate metabolism after being exposed to the MH4w condition; this finding suggests that hypoxia may affect the metabolism of Gansu zokors. Furthermore, our study is the first one to bin genomes from metagenomic datasets and obtain high-quality bins of *Bacteroidales*, *Clostridum*, and *Desulfovibrio*, their KEGG functional pathway information proves the role of these bacteria in affecting carbohydrate metabolism. Taken together, we showed that the SH44h or MH4w causes more pronounced changes in the composition and structure of gut microbiota in Gansu zokor. Furthermore, overall, hypoxia decreases the metabolism levels; a previous study also shows that energy metabolism is suppressed in the naked mole rat in response to chronic hypoxia (71); which indicates that low metabolism may represent an adaptive strategy for hypoxia tolerance.

### Gansu Zokors Enhance Glucose and Other Carbohydrate Fuel Substrates Utilization Under Moderate Hypoxia 4 Weeks Group Condition

As described previously, the KEGG pathway carbohydrate metabolism was the highest in the feces of Gansu zokor. Metagenomic sequencing in the carbohydrate metabolism gene set would expand our understanding of the carbohydrate metabolic pathway and be used for the analysis of the relationship between microbial function and host physiology (72, 73). In this study, we focused on one of the hypoxia groups, the MH4w treatment, which significantly altered the gut microbiota and dramatically reduced the carbohydrate metabolism level. Our results showed that after being exposed to the MH4w condition, the “carbohydrate transport and metabolism” was the most enriched functional COG category in Gansu zokor. The KEGG analysis further indicated that hypoxia increased glycolysis and inhibited the TCA cycle in Gansu zokor, which is consistent with other mammalian species in response to hypoxia (74). Independent of this, we found that the carbohydrate metabolism fuel in Gansu zokor strengthens the reliance on some substrates, such as fructose and mannose, suggesting that Gansu zokor has the capacity to initiate diverse substrates metabolism, especially under hypoxia. This carbohydrate metabolic pattern may contribute to the adaptations of the subterranean rodents to hypoxic environmental conditions. Moreover, it is rather interesting to note that the relative contribution of the *Ileibacterium* genus to carbohydrate metabolism in hypoxia condition of MH4w was far higher than that of its contribution in normoxia, particularly in glycolysis and some other substrate metabolism, such as fructose, which meant that the metagenomic plasticity of microbial symbionts under hypoxia may boost the ability to metabolism regulation. Furthermore, interestingly, the combined analysis of microbiome and carbohydrate metabolites shows that *Ileibacterium* and *Desulfovibrio* have the same correlation with differential metabolites. Overall, they are positively related to glycolysis products, such as phosphoenolpyruvic acid, but negatively related to TCA cycle products, such as citrate and succinate; moreover, they are also negatively related to ATP. These results indicate that the high abundance of *Ileibacterium* and *Desulfovibrio* might mediate the carbohydrate metabolism pattern by enhancing the glycolysis and attenuating the TCA cycle while decreasing the generated ATP *via* carbohydrate metabolism; this reveals the meaningfulness of increased abundance of *Ileibacterium* and *Desulfovibrio* after exposure to hypoxia of MH4w condition. Thus, metagenomic analysis and combined analysis results confirmed our initial hypothesis that the *Ileibacterium* genus could mediate the energy metabolism; this unique insight into the intestinal microflora of Gansu zokor was obtained in our study can be of great utility for further energy supply studies in hypoxia-tolerant subterranean rodents.

Alterations in the intestinal flora have profound influences on host metabolism (75); as the major metabolic organ, the liver can detect signals from the gut microbiome through the gut-liver axis and plays a crucial role in regulating metabolic homeostasis (76, 77). We, therefore, focused on the liver carbohydrate metabolism; the results showed that the hypoxia of MH4w treatment increased the metabolites of glycolysis, while it decreased the metabolites of the TCA cycle; besides, we also observed that this hypoxia may enhance the utilization of fructose and mannose in Gansu zokor liver. Results from the liver metabolite analyses preliminarily confirmed the results of the metagenomic analysis. In general terms, on the one hand, our study highlights once again the importance of intestinal microflora for regulating host metabolism; on the other hand, our findings suggest that Gansu zokor could change the intestinal flora under hypoxia, thereby mediating the utilization of carbohydrate fuel substrates; this metabolic remodeling seems to be an adaptive response to subterranean hypoxia.

### Gansu Zokors Reinforce Fructose-Driven Metabolism Capability Under Hypoxia, and This Species Attenuate Tricarboxylic Acid Cycle in the Liver Under Long-Term (4 Weeks) Hypoxia but Do Not Exhibit an Inhibition Under Short-Term (6 or 44 h) Hypoxia

Glucose and fructose are both substrates for the glycolytic pathway, playing important roles in carbohydrate metabolism (78). Unlike glucose, fructose is metabolized primarily by the liver and kidney (79, 80); however, some subterranean rodents have evolved the ability to utilize fructose to fuel the brain under hypoxia (9, 11), which may be the uniqueness of these species. Moreover, based on the findings of the aforementioned liver metabolites, we suspect that the subterranean hypoxia also could enhance liver fructose metabolism to provide energy rapidly in Gansu zokor, and this clearly merits research at the molecular level. Consistently, we found that Gansu zokor liver showed increased expression of metabolism-related fructose molecules in response to hypoxia stress. Beyond that, we also observed that the TCA cycle flux was reduced under long-term (4 weeks) hypoxia; however, such inhibition was not seen in other short-term hypoxia conditions. The current results demonstrated different adaptive strategies of Gansu zokor under various sets of hypoxic conditions.

The feedforward upregulation of fructolytic requires GLUT5 and KHK, which represent molecular signatures of fructose metabolism (81). It is interesting that although Gansu zokor increases the mRNA expression of *glut5* and *khk* only under some hypoxia conditions, this increasing phenomenon at their protein levels occurs in all hypoxic groups, which suggests that the substrates for fructose metabolism can improve the translation efficiency of these two key genes. In addition, the KHK activity was also significantly upregulated under hypoxia. These regulations can potentially enhance fructose metabolism rapidly in Gansu zokor liver under hypoxia, which might be one of the proactive strategies to enhance fast energy supply under hypoxia for this species. Furthermore, ALDOB and ALDOC are the common essential genes shared in fructose-driven and glucose-driven glycolysis pathways (40); in this study, we showed that their protein levels were increased in Gansu zokor liver under short-term (6 or 44 h) hypoxic conditions; the results are consistent with similar hypoxia responses in glycolysis reported in cancer cells (82). We then found that even with the increase in plasma fructose levels during two severe hypoxic conditions, concentrations of the fructose substrate in the liver decline under all hypoxia conditions, suggesting that hypoxia promotes fructose-driven glycolysis. Dietary fructose is the primary source of fructose (83), but obviously, the source of increased plasma fructose in Gansu zokor is not from dietary fructose intake; it probably results from endogenous fructose production (84, 85). Overall, Gansu zokors reinforce fructose-driven metabolism capability under hypoxia, thereby providing an adaptation strategy that allows for rapid energy supply in subterranean hypoxic environments.

Glucose is an important energy source, and its functions cannot be underestimated (86). The liver is the organ that can produce glucose and is a central platform for glucose metabolism in humans (87, 88). GLUT1 and GLUT2, the major glucose transmembrane transporters are present in the liver and serve as glucose sensors in regulating glucose uptake (89, 90). We found that GLUT2 protein levels were decreased in Gansu zokor liver under hypoxia condition; in contrast, we observed an increased hepatic expression of GLUT1 protein after being exposed to hypoxia; this phenomenon was more pronounced in SH44h and MH44h groups. These results indicated that in the hepatocytes, the hypoxia augmented the GLUT1-dependent glucose uptake and attenuated the GLUT2-dependent glucose uptake, which meant that different glucose transporters may play different roles in response to hypoxia. Furthermore, combined with genes expression analysis of other glucose-driven glycolysis and the enzymatic activities of PFK and PK, we found that apart from the MH4w group, Gansu zokor enhanced the above two key enzymatic activities and increased the overall protein levels of aldolases after being exposed to three short-term (6 or 44 h) hypoxic conditions. Our results indicate that the Gansu zokor liver has the capacity for increased glucose-driven glycolysis to cope with the short-term (6 or 44 h) hypoxia, which is consistent with the findings observed in the naked mole rat under hypoxia (4). Moreover, glucose was mobilized from the Gansu zokor liver to the blood in the three short-term (6 or 44 h) hypoxia groups, which also confirmed that Gansu zokor increased their reliance on glucose in hypoxia.

Under aerobic conditions, glucose is usually assumed to be fully burned by tissues through the TCA cycle (91). As evidenced by numerous studies, hypoxic conditions suppress the TCA cycle, such as it would occur in acute-on-chronic liver failure and cancer cells (92, 93). Citrate synthase initiates the TCA cycle and plays a critical role in this metabolism pathway (43, 94). On the one hand, our study showed that the CS enzymatic activity in the Gansu zokor liver was significantly downregulated under the MH4w condition, and the same results occurred in its mRNA and protein expression. These results indicated that similar to previous studies (92, 93), the long-term (4 weeks) moderate hypoxia would weaken the TCA cycle. Moreover, the above metabolite results showed that the levels of the TCA metabolites citrate and succinate were substantially reduced in the liver following MH4w hypoxia, which also provides evidence that the TCA cycle is inhibited under long-term (4 weeks) moderate hypoxia. On the other hand, interestingly, the hypoxia of short-term (6 or 44 h) treatments increased the activities of three key metabolic enzymes in the TCA cycle, indicating that the short-term hypoxia strengthens the TCA cycle, which is contrary to the results from previous studies on the hypoxia and its metabolism. These findings indicated that Gansu zokors appeared to use a different metabolic pattern to suit different hypoxia stress. Lastly, we also found that at the oxygen concentration of 6.5%, the carbohydrate metabolism in the Gansu zokor liver was enhanced with the extension of time; but this metabolism was reduced with the prolongation of treatment time at the oxygen concentration of 10.5%. Taken together, our study observed an enhancement of carbohydrate metabolism in Gansu zokors which helps them to survive under the short-term (6 or 44 h) hypoxia, especially in severe hypoxia, and similar to naked mole rats (71), Gansu zokors suppress energy metabolism in the long-term moderate hypoxia, thereby reducing the oxygen demand and this makes them successfully survive in their subterranean hypoxic environments.

## Study Limitations

There are some limitations to this study. First, our selection of the low oxygen concentrations (6.5% O_2_ or 10.5% O_2_) was mainly based on hypoxia tolerance tests in Gansu zokor and the previous studies of *Spalax* (48, 95); further studies should collect the oxygen concentration of the wild subterranean burrows of Gansu zokors at different depths, weather, and seasons. Second, the sample size of Gansu zokors in this study was relatively small; thus, the persuasiveness of the obtained results may be limited, and a larger number should be assessed in future studies. Besides, more evidence is needed to verify the function of the gut microbiome; therefore, fecal microbiota transplantation and fecal metabolite profiling will be required to make up for the insufficient evidence in the following research. Finally, in addition to carbohydrate metabolism, how the gut microbiome attributes to other metabolism pathways, immune system, cancer, and aging in Gansu zokors is worthy of further investigation.

## Conclusion

Our study offers a comprehensive account of the fecal microbiota of Gansu zokor under hypoxia as the first report. The microbial composition undergoes a reorganization in this species after being exposed to hypoxia, thus mediating the carbohydrate metabolism pattern, which seems to be an adaptive regulation in response to the subterranean hypoxic environment. We believe that the composition and structure of the gut microbiota under hypoxia in the subterranean hypoxia-tolerant animals is an important resource, which will open a new facet for hypoxic damage therapeutics and biotechnology applications. Furthermore, the metabolic flexibility of the Gansu zokor liver can mediate different carbohydrate metabolic patterns under different hypoxia conditions, providing this species with a strong advantage in the face of dynamic subterranean hypoxic environments to meet bioenergetic needs. These changes in the liver carbohydrate metabolic pathways under hypoxia were consistent with the above changes in the functional capacity of the microbiota. In general, our study reveals the mechanism of the metabolic adaptation underlying hypoxia tolerance in Gansu zokor, which suggests a novel strategy for human hypoxic disease therapy.

## Data Availability Statement

The original contributions presented in this study are publicly available. This data can be found here: NCBI, PRJNA796738, PRJNA797577.

## Ethics Statement

The animal study was reviewed and approved by Animal Management Committee and Ethical Review Committee of Experimental Animal Welfare, Shaanxi Normal University.

## Author Contributions

JGL and JH conceived and designed the study. JYL wrote the manuscript with the help of JGL. JYL and QY collected the samples and performed the experimental work. JG, ML, and ZH analyzed the data. All authors contributed to the revision of manuscript and approved the final manuscript.

## Conflict of Interest

The authors declare that the research was conducted in the absence of any commercial or financial relationships that could be construed as a potential conflict of interest.

## Publisher’s Note

All claims expressed in this article are solely those of the authors and do not necessarily represent those of their affiliated organizations, or those of the publisher, the editors and the reviewers. Any product that may be evaluated in this article, or claim that may be made by its manufacturer, is not guaranteed or endorsed by the publisher.

## Footnotes

1 http://drive5.com/uparse/, version 7.1

2
http://rdp.cme.msu.edu/

3
https://www.arb-silva.de/

4
https://github.com/najoshi/sickle

5
https://github.com/ShujiaHuang/SOAPaligner

6
http://metagene.nig.ac.jp

7
http://www.genome.jp

8 https://github.com/BinPro/CONCOCT, version 0.5.0

9 https://bitbucket.org/berkeleylab/metabat, version 2.12.1

10
https://github.com/Ecogenomics/CheckM/wiki

11
https://github.com/martinwu/AMPHORA2

12
https://cloud.majorbio.com/

13
http://picrust.github.io/picrust/

14
https://github.com/picrust/picrust2/
