# Supplementary material for: Gut Microbiome Alterations and Hepatic Metabolic Flexibility in the Gansu Zokor, Eospalax cansus: Adaptation to Hypoxic Niches
Source: Front Cardiovasc Med. 2022 Mar 23;9:814076. doi: 10.3389/fcvm.2022.814076 (PMC8984292; doi:10.3389/fcvm.2022.814076)
Supplement: Supplementary file 1 [file Data_Sheet_1.pdf]

Supplementary materials

1. Supplementary methods

1.1 RNA extraction and quantitative real-time PCR verification

Total RNA was extracted from liver tissues using TRIzol reagent (TaKaRa, Beijing, China) according to the manufacturer's instructions. The RNA samples were then reverse transcribed to cDNA using a Prime Script II 1st Strand cDNA Synthesis kit (TaKaRa, Beijing, China). The primers sequences were listed in Table S1. Six technical replicates were prepared for the analysis of each gene by quantitative real-time PCR verification (qRT-PCR) using a Step One Real-Time System (ABI). The samples were analyzed in a 25 μl reaction volume, which consisted of 12.5 μl SYBRR Premix Ex TaqII (Tli RNaseH Plus) (2×), 1 μl each primer (10 μM), 8.5 μl nuclease-free water, and 2 μl template cDNA. The relative quantifications of genes expressions were measured using CFX96TM Real-time Detection System (Bio-Rad). Beta-actin (bactin) mRNA levels were used to normalize the levels of target genes via the $2^{-\Delta\Delta Ct}$ method.

1.2 Western-blotting

Liver samples were pulverized and transferred to a separate tube with RIPA buffer for 35 min on ice, then the protein samples were collected after centrifugation (13,000 *rpm*, for 30 min at 4 ℃) and protein concentrations were measured from the resulting supernatants using a BCA protein assay kit from Nanjing Jiancheng Bioengineering Institute (Nanjing, China). Samples containing an equal amount of protein (20 μg per well) were separated by SDS-PAGE at appropriate acrylamide concentrations and transferred to PVDF membranes. After blocking in 5% skim milk for 2 h on the shaker at room temperature, the membranes were incubated overnight at 4℃ with primary antibodies of GLUT1 (1:40,000; Abcam, Cambridge, MA, United States), GLUT2 (1:300; ProteinTech Group, Chicago, IL, United States), GLUT5 (1:500; Affinity Biosciences, Cincinnati, OH, United States), PFK (1:5,000; Abcam, Cambridge, MA, United States), KHK (1:2,500; Abcam, Cambridge, MA, United States), ALDOA (1:10,000; ProteinTech Group, Chicago, IL, United States), ALDOB (1:8,000; ProteinTech Group, Chicago, IL, United States), ALDOC (1:5,000; ProteinTech Group, Chicago, IL, United States), PKLR (1:500; ProteinTech Group, Chicago, IL, United States), CS (1:10,000; Abcam, Cambridge, MA, United States) and β-actin (1:16,000; ABclonal, Wuhan, China); followed by incubation with the secondary anti-rabbit IgG conjugated (1:3,000; ABclonal, Wuhan, China) at room temperature for 2 h. Visualization was implemented using the ECL kit (Pierce, Rockford, United States) according to the manufacturer's instructions. Quantification was carried out using Image J Software.

1.3 Liver metabolite profiling

A total of six liver samples (Norm group: 2 females and 1 males; MH4w group: 2 females and 1 males) were selected for metabolite profiling. Approximately 50 mg of samples were placed into 500 μl pre-cooled extractant (70% methanol aqueous solution), homogenized and centrifuged to take 200 μl of supernatant for LC-MS/MS

analysis using an LC-ESI-MS/MS system (UPLC, Shim-pack UFLC SHIMADZU CBM30A system, https://www.shimadzu.com/; MS, QTRAP® System, https://sciex.com/). The analytical conditions were as follows, UPLC: column, SeQuant ZIC-pHILIC 5 μm (100 mm × 2.1 mm); column temperature, 40 ℃; flow rate, 0.4 ml/min; solvent system, 10 mmol/L ammonium acetate + 0.3% ammonia solution, 90% acetonitrile water; gradient program, 5:95 V/V at 0 min, 50:50 V/V at 9.5 min, 5:95 V/V at 11.1 min, 5:95 V/V at 14.0 min. The injection volume for each samples was 2 μl.

A triple quadrupole-linear ion trap mass spectrometer (QTRAP) was used to acquire LIT and triple quadrupole (QQQ) scans. The QTRAP was operated in both positive and negative ion modes and controlled by Analyst 1.6.3 software (Sciex). The curtain gas, ion source gas I and ion source gas II were set at 40, 55 and 35 PSI, respectively; and the source temperature was 450 ℃. The ion spray voltage was set at 5500 V (positive) and -4500 V (negative). Instrument tuning and mass calibration were performed with 10 and 100 μmol/L polypropylene glycol solutions in QQQ and LIT modes, respectively. A specific set of MRM transitions were monitored for each period according to the metabolites eluted within this period.

1.4 Enzymatic activity assay

The phosphate buffer saline (10 times the volume of tissue) was added in liver samples for homogenization. Then the supernatant samples were collected after centrifugation (5,000 *rpm*, for 15 min at room temperature) and enzymatic activities of PFK, KHK, PK, CS, IDH and α-KGDHC were measured using the enzyme-linked immunoassay (ELISA) kits (PFK kit, Kete, Yancheng, China; KHK kit, Meimian, Yancheng, China; PK kit, Kete, Yancheng, China; CS kit, Meimian, Yancheng, China; IDH kit, Meimian, Yancheng, China; α-KGDHC kit, Meimian, Yancheng, China), according to the manufacturers' instructions.

1.5 Glucose and fructose determination

Approximately 0.1 g liver tissue was placed into 1 ml dd H_2O, homogenized and centrifuged to collect the supernatant (8,500 *rpm*, for 10 min at room temperature). The glucose and fructose contents in supernatant samples and plasma were assessed using glucose (Feiya, Yancheng, China) and fructose (Feiya, Yancheng, China) content kits according to the manufacturers' instructions.

1.6 Statistical analysis

The qPCR, western blot, enzymatic activity, glucose and fructose measurements were analysed using ANOVA followed by Tukey's *post hoc* tests. P-values < 0.05 were considered to indicate statistical significance. For liver metabolomic analysis, an unsupervised principal component analysis (PCA) was performed to obtain the relationships among the data matrix. VIP values were extracted from orthogonal partial least squares discriminate analysis (OPLS-DA). Differential changed metabolites were identified when the VIP values > 1.0 and fold change > 1.2 (or < 0.83). Identified metabolites were annotated and mapped to KEGG pathway database (http://www.kegg.jp/kegg/compound/ and http://www.kegg.jp/kegg/pathway.html). Significance was determined by hypergeometric test's P-values.

2. Supplementary figures

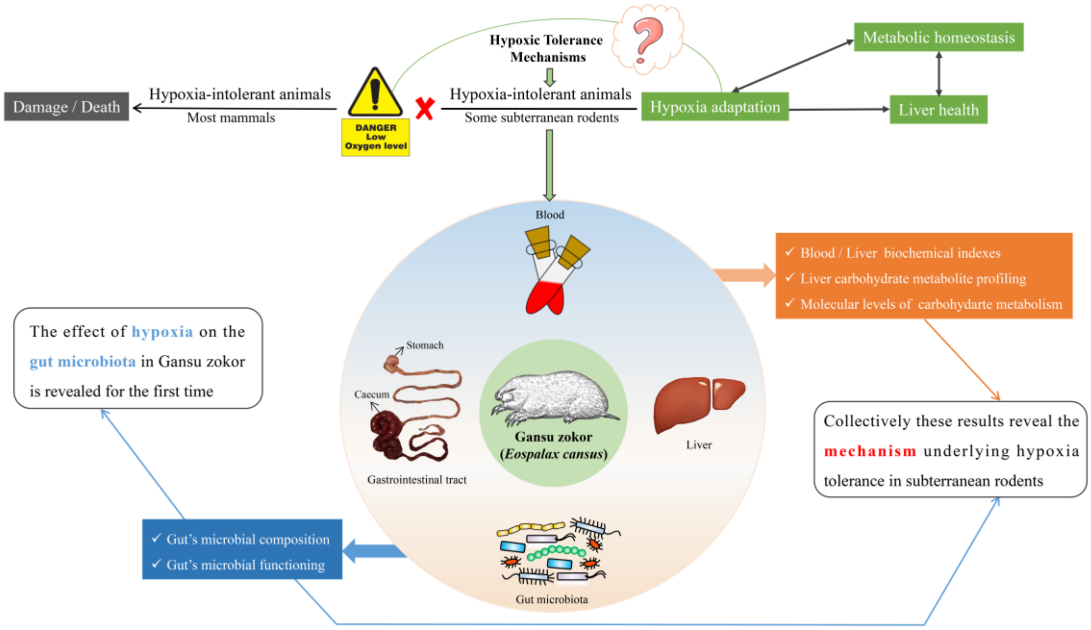

Figure S1. The key research ideas and experimental protocols. Intestinal microflora, physiological, liver metabolism variables in Gansu zokors under normoxia and hypoxia were comprehensively evaluated.

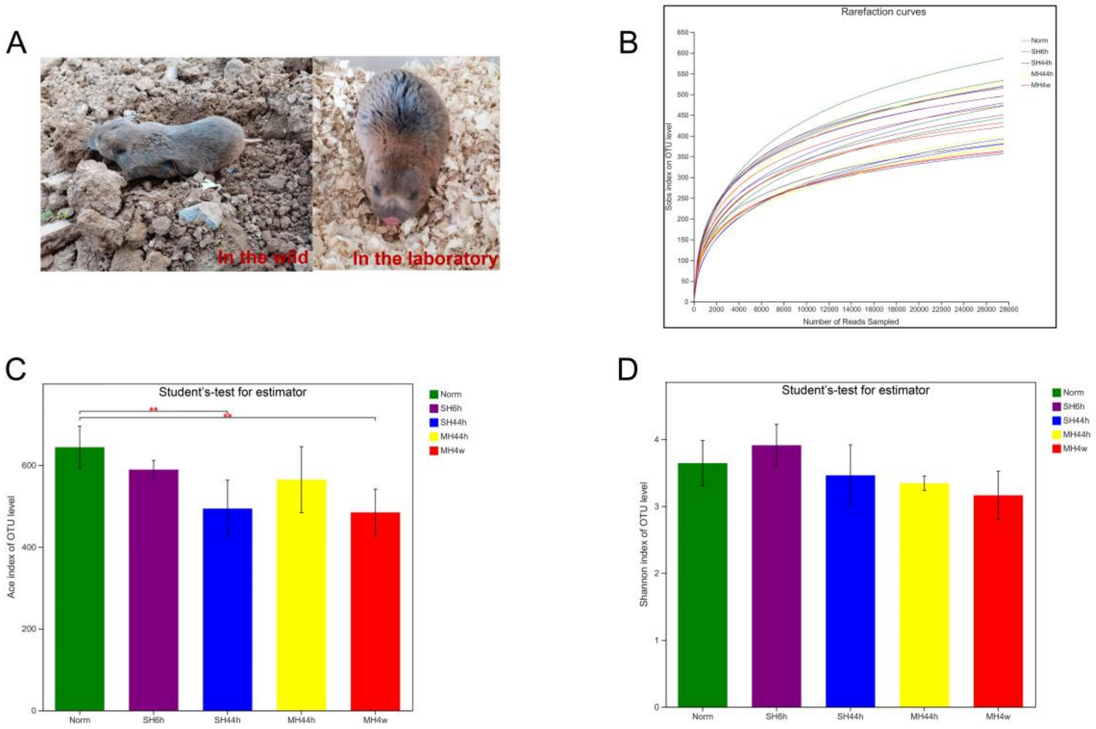

Figure S2. Rarefaction curves and Alpha-diversity of gut microbiota of Gansu zokors **(A)** in different groups. **(B)** Rarefaction curves of all the samples based on Illumina MiSeq sequencing. Horizontal axis: The effective number of sequences of samples; vertical axis: the observed richness (Sobs) at the OUT level. The richness of the sample is estimated by the richness index Sobs. **(C)** Community richness index: ACE index and **(D)** Community biodiversity index: Shannon index on OUT level. Statistical symbols: ** $P < 0.01$.

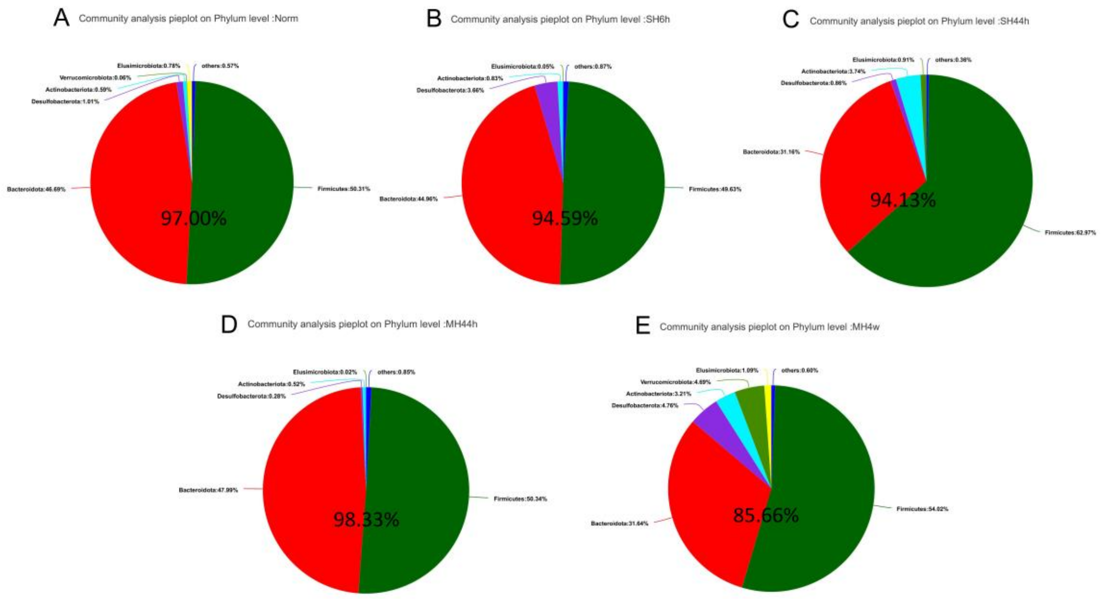

Figure S3. Proportion of dominant phyla. The top two phyla of Firmicutes and Bacteroidota in Gansu zokors account for 97 % in normoxia **(A)**, 94.59 % in SH6h **(B)**, 94.13 % in SH44h **(C)**, 98.33 % in MH44h **(D)** and 85.66 % in MH4w **(E)** of the total sequences.

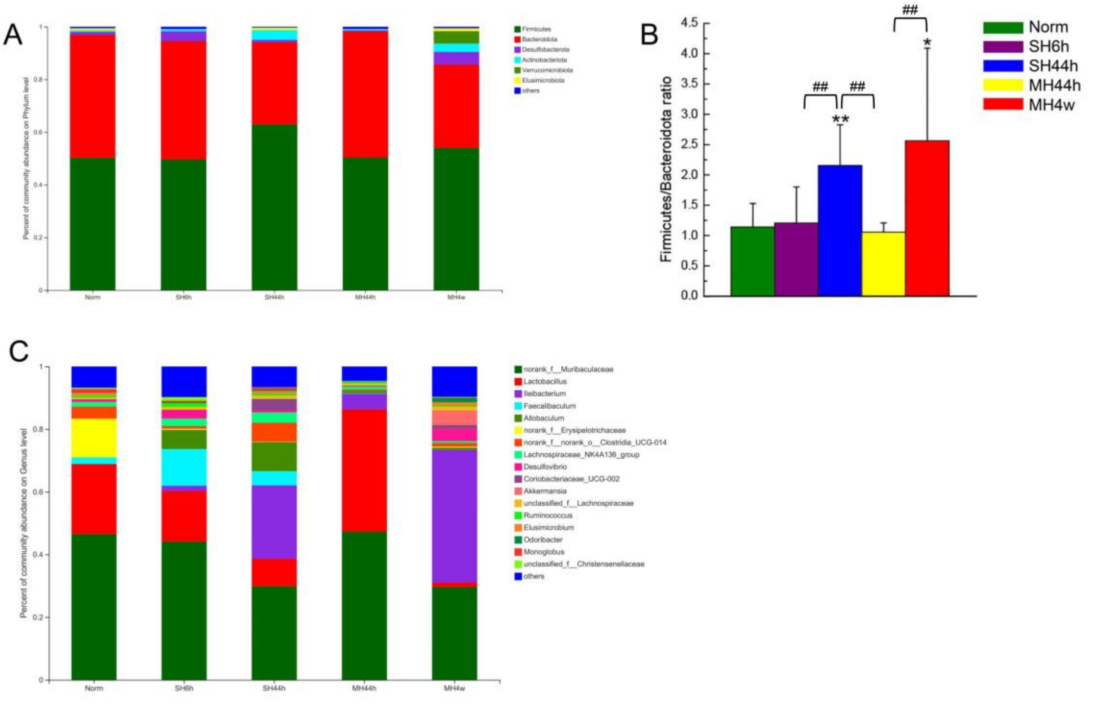

Figure S4. Relative abundances of dominant microbial taxa. **(A)** Relative abundances of dominant microbial phyla. **(B)** The ratio of Firmicutes/Bacteroidota. **(C)** Relative abundances of dominant microbial genera. Statistical symbols: * $P < 0.05$, ** $P < 0.01$, and ## $P < 0.01$.

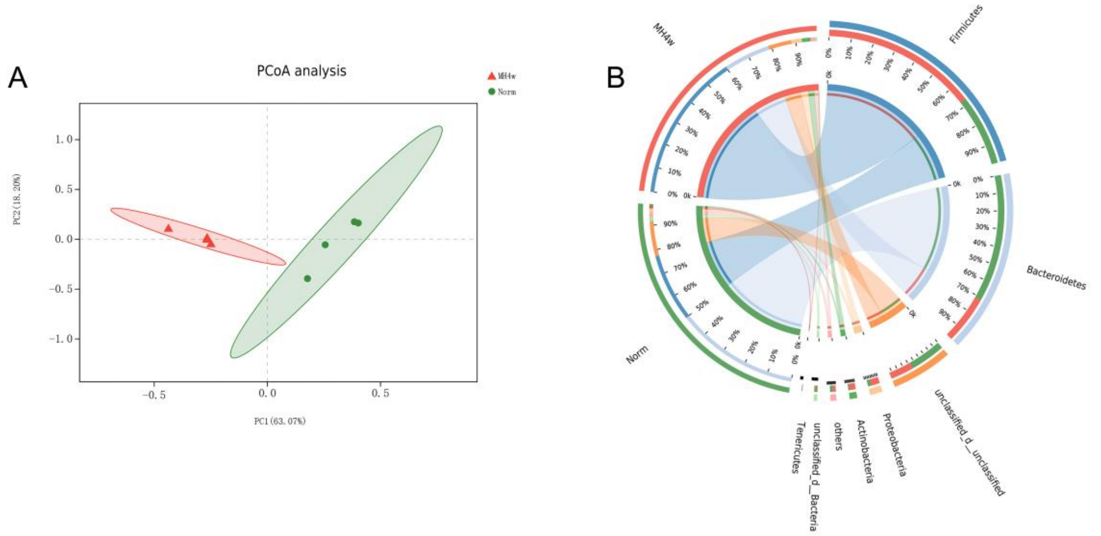

Figure S5. Principal component analysis (PCoA) of microbial communities from normoxia and MH4w **(A)**, and Circos plot of relationship between two groups and their microbioal phyla **(B)**.

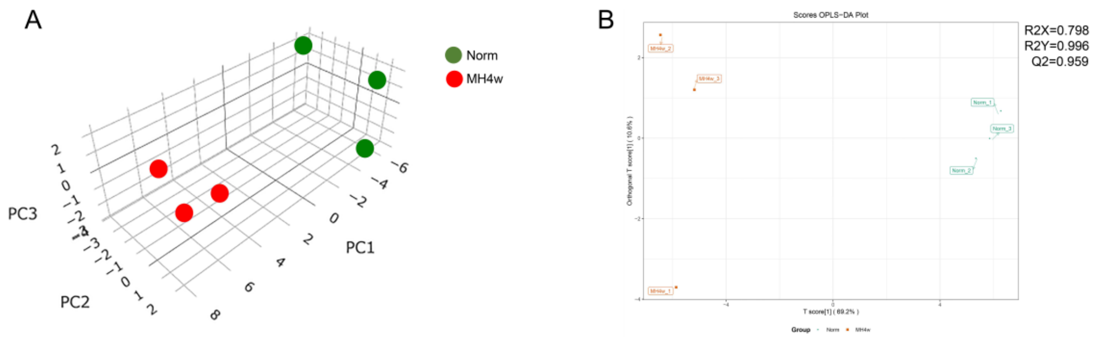

Figure S6. The metabolic profiles of liver samples. Principal component analysis (PCA) **(A)** and partial least square discriminant analysis (PLS-DA) score plots **(B)** for comprehensive metabolites data.

3. Supplementary tables

Table S1. The average percentage of Verrucomicrobiota and *Akkermansia*.

Species name	Norm	SH6h	SH44h	MH44h	MH4w
Verrucomicrobiota	0.05952%	0%	0%	0%	4.685%
Akkermansia	0.05952%	0%	0%	0%	4.685%

Table S2 | The 89 binned genomes from metagenomic-combined assembly.

Bin ID	Genome size (bp)	Completeness (%)	Contamination (%)	Taxonomy
bin205	2311306	99.25	1.75	d_Bacteria;p_Firmicutes;c_Bacilli;o_Lactobacillales;f_Streptococcaceae;g_*Streptococcus*
bin129	2706939	99.06	7.94	d_Bacteria;p_Bacteroidetes;c_Bacteroidia;o_Bacter

				oidales
bin118	2138083	98.49	0.57	d_Bacteria;p_Bacteroidetes;c_Bacteroidia;o_Bacteroidales
bin16	2334043	98.11	7.95	d_Bacteria
bin352	2837053	98.05	1.71	d_Bacteria
bin176	2558127	97.87	2.17	d_Bacteria;p_Bacteroidetes;c_Bacteroidia;o_Bacteroidales
bin308	2145348	97.74	0.67	d_Bacteria;p_Bacteroidetes
bin203	2346259	97.61	6.6	d_Bacteria;p_Bacteroidetes;c_Bacteroidia;o_Bacteroidales
bin290	1809777	97.6	0	d_Bacteria
bin335	2184446	97.5	6.21	d_Bacteria;p_Actinobacteria;c_Actinobacteria;o_Bifidobacteriales;f_Bifidobacteriaceae;g_*Bifidobacterium*;s_*Bifidobacterium animalis*
bin264	1837036	97.48	0.83	d_Bacteria
bin380	2907521	97.29	3.47	d_Bacteria
bin406	2541521	96.67	2.08	d_Bacteria;p_Bacteroidetes
bin33	2010844	96.64	0.67	d_Bacteria;p_Firmicutes;c_Clostridia;o_Clostridiales
bin326	2310032	96.6	3.71	d_Bacteria;p_Bacteroidetes;c_Bacteroidia;o_Bacteroidales
bin95	1559556	96.59	0.89	d_Bacteria;p_Actinobacteria;c_Actinobacteria;o_Coriobacteriales;f_Coriobacteriaceae
bin417	3016588	96.45	3.91	d_Bacteria
bin282	3044896	96.31	3.82	d_Bacteria;p_Firmicutes;c_Clostridia;o_Clostridiales
bin109	1906807	96.04	2.82	d_Bacteria;p_Firmicutes;c_Clostridia;o_Clostridiales
bin201	2451569	95.97	0.75	d_Bacteria;p_Bacteroidetes
bin312	2660677	95.26	0.4	d_Bacteria
bin207	2403502	95.09	2.58	d_Bacteria;p_Bacteroidetes
bin261	1776325	94.36	0	d_Bacteria;p_Firmicutes
bin111	2099177	94.09	6.42	d_Bacteria;p_Bacteroidetes
bin369	2338593	93.99	1.32	d_Bacteria;p_Bacteroidetes;c_Bacteroidia;o_Bacteroidales
bin105	3125639	93.94	2.01	d_Bacteria;p_Bacteroidetes
bin124	2189395	93.77	2.26	d_Bacteria;p_Bacteroidetes;c_Bacteroidia;o_Bacter

				oidales
bin398	2324950	93.77	1.15	d_Bacteria;p_Bacteroidetes;c_Bacteroidia;o_Bacteroidales
bin318	2130524	93	4.22	d_Bacteria;p_Proteobacteria;c_Deltaproteobacteria;o_Desulfovibrionales;f_Desulfovibrionaceae
bin60	2465013	92.9	3.32	d_Bacteria;p_Proteobacteria;c_Deltaproteobacteria;o_Desulfovibrionales;f_Desulfovibrionaceae
bin344	2687626	92.77	7.37	d_Bacteria;p_Bacteroidetes
bin255	1789811	92.59	0.89	d_Bacteria;p_Firmicutes;c_Clostridia;o_Clostridiales
bin212	2096512	92.53	2.79	d_Bacteria
bin163	2343783	92.5	7.84	d_Bacteria;p_Bacteroidetes;c_Bacteroidia;o_Bacteroidales
bin149	1736384	92.44	0.67	d_Bacteria;p_Firmicutes;c_Clostridia;o_Clostridiales
bin27	2411916	92.18	3.87	d_Bacteria;p_Proteobacteria;c_Deltaproteobacteria;o_Desulfovibrionales
bin3	2094792	91.91	2.39	d_Bacteria;p_Bacteroidetes;c_Bacteroidia;o_Bacteroidales
bin225	2703639	91.7	2.16	d_Bacteria
bin189	2149945	91.37	3.75	d_Bacteria;p_Bacteroidetes
bin247	3015824	91.15	4.01	d_Bacteria;p_Bacteroidetes
bin389	1643643	90.76	4.48	d_Bacteria;p_Firmicutes;c_Bacilli;o_Lactobacillales;f_Lactobacillaceae;g_*Lactobacillus*
bin9	1621036	90.5	3.32	d_Bacteria;p_Firmicutes;c_Clostridia;o_Clostridiales
bin11	1782687	90.18	3.8	d_Bacteria;p_Firmicutes;c_Clostridia
bin116	1810646	89.44	2	d_Bacteria
bin75	1154848	89.33	0	d_Bacteria
bin409	2587524	89.23	3.21	d_Bacteria;p_Bacteroidetes;c_Bacteroidia;o_Bacteroidales
bin422	2334898	88.74	8.84	d_Bacteria;p_Bacteroidetes;c_Bacteroidia;o_Bacteroidales
bin419	2011245	88.61	2.86	d_Bacteria;p_Proteobacteria;c_Deltaproteobacteria;o_Desulfovibrionales;f_Desulfovibrionaceae
bin152	2671963	88.18	1.51	d_Bacteria;p_Bacteroidetes;c_Bacteroidia;o_Bacteroidales
bin150	2150958	87.63	1.52	d_Bacteria;p_Bacteroidetes;c_Bacteroidia;o_Bacteroidales
bin107	2367222	87.33	4.7	d_Bacteria;p_Proteobacteria;c_Deltaproteobacteria;

				o_Desulfovibrionales;f_Desulfovibrionaceae
bin387	2312441	87.29	4.84	d_Bacteria;p_Bacteroidetes;c_Bacteroidia;o_Bacteroidales
bin376	2089886	87.1	0	d_Bacteria;p_Bacteroidetes;c_Bacteroidia;o_Bacteroidales;f_Porphyromonadaceae;g_*Odoribacter*;s_*Odoribacter splanchnicus*
bin400	2204260	87.04	3.96	d_Bacteria
bin273	2391448	86.04	0.5	d_Bacteria;p_Bacteroidetes;c_Bacteroidia;o_Bacteroidales
bin136	1756080	85.69	0.38	d_Bacteria;p_Bacteroidetes;c_Bacteroidia;o_Bacteroidales
bin52	2978978	85.22	4.89	d_Bacteria;p_Firmicutes;c_Clostridia;o_Clostridiales
bin148	1670602	85.1	7.13	d_Bacteria;p_Firmicutes;c_Clostridia
bin283	2195654	84.29	6.09	d_Bacteria;p_Bacteroidetes;c_Bacteroidia;o_Bacteroidales
bin306	2035278	83.76	4.07	d_Bacteria;p_Bacteroidetes;c_Bacteroidia;o_Bacteroidales
bin114	1680715	81.46	0	d_Bacteria;p_Spirochaetes;c_Spirochaetia;o_Spirochaetales;f_Spirochaetaceae;g_*Sphaerochaeta*
bin80	2549467	81.13	2.29	d_Bacteria
bin218	1731462	81.03	3.76	d_Bacteria;p_Bacteroidetes;c_Bacteroidia;o_Bacteroidales
bin34	2401874	80.58	5.3	d_Bacteria
bin144	1784719	80.57	3.96	d_Bacteria;p_Bacteroidetes;c_Bacteroidia;o_Bacteroidales
bin194	2014271	79.93	6.16	d_Bacteria;p_Bacteroidetes;c_Bacteroidia;o_Bacteroidales
bin180	2071761	78.81	0.19	d_Bacteria;p_Bacteroidetes;c_Bacteroidia;o_Bacteroidales
bin101	1969526	78.11	0.78	d_Bacteria;p_Bacteroidetes;c_Bacteroidia;o_Bacteroidales
bin172	2142656	77.92	4.62	d_Bacteria;p_Bacteroidetes
bin425	1962256	77.42	7.53	d_Bacteria;p_Bacteroidetes;c_Bacteroidia;o_Bacteroidales;f_Porphyromonadaceae;g_*Odoribacter*;s_*Odoribacter splanchnicus*
bin397	1986554	76.46	2.83	d_Bacteria;p_Bacteroidetes;c_Bacteroidia;o_Bacteroidales
bin137	1990003	76.39	1.48	d_Bacteria;p_Bacteroidetes;c_Bacteroidia;o_Bacteroidales
bin43	1451093	76.19	1.17	d_Bacteria;p_Firmicutes;c_Clostridia;o_Clostridial

				es
bin317	2469573	75.35	0.72	d_Bacteria;p_Firmicutes;c_Clostridia;o_Clostridiales
bin133	1796785	74.68	2.26	d_Bacteria;p_Bacteroidetes;c_Bacteroidia;o_Bacteroidales
bin252	1825473	74.55	5.47	d_Bacteria;p_Bacteroidetes;c_Bacteroidia;o_Bacteroidales
bin379	2415674	73.71	1.28	d_Bacteria
bin256	2282900	73.54	8.62	d_Bacteria;p_Bacteroidetes;c_Bacteroidia;o_Bacteroidales
bin168	1345612	73.42	1.73	d_Bacteria
bin88	1870370	73.19	5.35	d_Bacteria
bin54	1718916	72.92	3.33	d_Bacteria;p_Bacteroidetes;c_Bacteroidia;o_Bacteroidales
bin284	1730212	72.26	0.75	d_Bacteria;p_Bacteroidetes;c_Bacteroidia;o_Bacteroidales
bin418	1847834	72.24	0.54	d_Bacteria
bin316	1797272	72.24	0.85	d_Bacteria;p_Bacteroidetes;c_Bacteroidia;o_Bacteroidales
bin328	1895670	71.89	1.76	d_Bacteria;p_Bacteroidetes;c_Bacteroidia;o_Bacteroidales
bin315	1808546	71.03	0	d_Bacteria;p_Bacteroidetes;c_Bacteroidia;o_Bacteroidales
bin234	1104025	70.84	1.81	d_Bacteria;p_Firmicutes;c_Clostridia
bin286	1589242	70.69	0	d_Bacteria;p_Bacteroidetes;c_Bacteroidia;o_Bacteroidales
bin26	1805054	70	1.66	d_Bacteria;p_Bacteroidetes;c_Bacteroidia;o_Bacteroidales

Table S3 | The high-quality *Bacteroidales*, *Clostridum* and *Desulfovibrio*-related bins (≥ 90% complete and ≤ 5 % contamination).

Bin ID	Genome size (bp)	Completeness (%)	Contamination (%)	Taxonomy
bin118	2138083	98.49	0.57	d_Bacteria;p_Bacteroidetes;c_Bacteroidia;o_Bacteroidales
bin176	2558127	97.87	2.17	d_Bacteria;p_Bacteroidetes;c_Bacteroidia;o_Bacteroidales
bin326	2310032	96.6	3.71	d_Bacteria;p_Bacteroidetes;c_Bacteroidia;o_Bacteroidales
bin369	2338593	93.99	1.32	d_Bacteria;p_Bacteroidetes;c_Bacteroidia;o_Bacteroidales

bin124	2189395	93.77	2.26	d_Bacteria;p_Bacteroidetes;c_Bacteroidia;o_Bacteroidales
bin398	2324950	93.77	1.15	d_Bacteria;p_Bacteroidetes;c_Bacteroidia;o_Bacteroidales
bin3	2094792	91.91	2.39	d_Bacteria;p_Bacteroidetes;c_Bacteroidia;o_Bacteroidales
bin33	2010844	96.64	0.67	d_Bacteria;p_Firmicutes;c_Clostridia;o_Clostridiales
bin282	3044896	96.31	3.82	d_Bacteria;p_Firmicutes;c_Clostridia;o_Clostridiales
bin109	1906807	96.04	2.82	d_Bacteria;p_Firmicutes;c_Clostridia;o_Clostridiales
bin255	1789811	92.59	0.89	d_Bacteria;p_Firmicutes;c_Clostridia;o_Clostridiales
bin149	1736384	92.44	0.67	d_Bacteria;p_Firmicutes;c_Clostridia;o_Clostridiales
bin9	1621036	90.5	3.32	d_Bacteria;p_Firmicutes;c_Clostridia;o_Clostridiales
bin318	2130524	93	4.22	d_Bacteria;p_Proteobacteria;c_Deltaproteobacteria;o_Desulfovibrionales;f_Desulfovibrionaceae
bin60	2465013	92.9	3.32	d_Bacteria;p_Proteobacteria;c_Deltaproteobacteria;o_Desulfovibrionales;f_Desulfovibrionaceae

Table S4 | Assembly results of 3 high-quality geonomes.

Genome	Bin Name	G+C (%)	Bin coverage	Genome coverage	Taxonomy
G_bin118	Bin118	52.05 %	26	20	Bacteroidales
G_bin33	bin33	40.43 %	48	35	Clostridiales
G_bin318	bin318	51.08 %	23	18	Desulfovibrionaceae

Table S5. Identified differential metabolites in Normoxia vs. MH4w group.

Compounds	Class	VIP	Fold_Change	Log2FC	Type
L-Serine	Amino Acid metabolomics	1.1691542	1.201928	0.2653503	up
L-Alanine	Amino Acid metabolomics	1.1139240	1.4826667	0.5681943	up
Glyoxylate	Amino Acid metabolomics	1.1061986	1.4948088	0.5799609	up
D-Glutamine	Amino Acid metabolomics	1.1508631	1.2207097	0.2877201	up
argininosuccinic acid	Amino Acid metabolomics	1.0554100	1.2572739	0.3302990	up
L-citrulline	Amino Acid metabolomics	1.1199387	1.6085946	0.6858008	up
Glucose	Carbohydrate metabolomics	1.1857712	1.2779230	0.3538009	up

Phosphoenolpyruvic acid	Carbohydrate metabolomics	1.1501624	1.5344093	0.6176834	up
Dihydroxyacetone phosphate	Carbohydrate metabolomics	1.1834989	1.5203909	0.6044423	up
Xyluose-5-phosphate	Carbohydrate metabolomics	1.189713	1.3559337	0.4392866	up
Inosine	Nucleotide metabolomics	1.1529734	1.2676569	0.3421644	up
Guanosine	Nucleotide metabolomics	1.1049501	1.2739063	0.3492591	up
L-Lactate	Organic Acid And Its Derivatives	1.1603924	1.2873724	0.3644294	up
2-Phospho-D-glyceric acid	Others	1.1969531	1.6485513	0.7211987	up
Glyceraldehyde 3-phosphate	Others	1.1796702	1.4997057	0.5846795	up
L-Asparagine	Amino Acid metabolomics	1.1885999	0.6743966	-0.5683308	down
Succinate/Succinic acid	Amino Acid metabolomics	1.1116771	0.7837912	-0.3514588	down
Citrate/Citric acid	Amino Acid metabolomics	1.1165903	0.7302024	-0.4536316	down
L-Glutamate	Amino Acid metabolomics	1.2012186	0.7301991	-0.4536382	down
D-Glucose 1-phosphate	Carbohydrate metabolomics	1.0986551	0.8236388	-0.2799162	down
sedoheptulose 7-phosphate	Carbohydrate metabolomics	1.179464	0.7058556	-0.502555	down
Acetyl-CoA	Coenzyme	1.1226232	0.7267204	-0.4605277	down
Thiamine pyrophosphate (TPP)	Co-Enzyme Factor & vitamin	1.1812862	0.5421068	-0.8833510	down
NAD+	Nucleotide metabolomics	1.0606819	0.8053853	-0.3122489	down
dTMP	Nucleotide metabolomics	1.1817187	0.6752588	-0.5664875	down
UDP-GlcNAc	Nucleotide metabolomics	1.1556024	0.7680507	-0.3807266	down
ATP	Nucleotide metabolomics	1.1121234	0.7880499	-0.3436412	down
Guanosine diphosphate (GDP)	Nucleotide metabolomics	1.1536317	0.8136344	-0.2975474	down
Glycerol 3-phosphate	Others	1.1749044	0.7933734	-0.3339280	down

Table S6 | Primers for qRT-PCR analysis.

Gene	Primer	Primer sequence (5′-3′)	Tm (℃)
glut1	Forward	GTCCCTACGTCTTCATCATCTT	58
	Reverse	CCGGAAGCCAGAAGCAATCTC	
glut2	Forward	TCCTTTGGTTTCTGGCACT	58
	Reverse	TTCCAGTACATCGCGGACTT	
glut5	Forward	GAACGATCTGGCCTTGGTCT	57
	Reverse	TTCATGGTCGGAGGCAGTGT	

pfk	Forward	AGATAGCCGCAGTAACCAC	59
	Reverse	CTCAATACCATCTGCACGACT	
khk	Forward	TAGAACAGCACAATGACGGG	60
	Reverse	GACAAACACCACCTCGCCAT	
aldoa	Forward	AGGCTCGCTTGATGTACTCC	58
	Reverse	CCTCAATGCCATCAACAAGTGC	
aldob	Forward	ATGCCACTCTCAATCTCAATGC	58
	Reverse	CTCCTGGGTTGCCTTCTTGTT	
aldoc	Forward	TCTCAGGCTCCACAATAGGCAC	58
	Reverse	TTTGCTAAATGGCGCTGTGTC	
pk	Forward	CTCATCTGTACCGTGGCATCTT	59
	Reverse	AGTTCACACGGAGGTCTACAT	
cs	Forward	CCCAAGATACCTGTTCCTCTG	59
	Reverse	AAGGCTAAGGGTGGGAAGAA	
β-actin	Forward	CTAAGGCCAACCGTGAAAAGAT	60 ±5
	Reverse	GACCAGAGGCATACAGGGACA	
